# High-fidelity *de novo* synthesis of pathways using microchip-synthesized oligonucleotides and general molecular biology equipment

**DOI:** 10.1038/s41598-017-06428-0

**Published:** 2017-07-21

**Authors:** Wen Wan, Min Lu, Dongmei Wang, Xiaolian Gao, Jiong Hong

**Affiliations:** 1 School of Life Sciences, University of Science and Technology of China, Hefei, Anhui 230027 China; 2 The Key Laboratory of Biotechnology for Medicinal Plant of Jiangsu Province, Jiangsu Normal University, Xuzhou, Jiangsu 221116 China; 3 Department of Biology and Biochemistry, University of Houston, Houston, TX77004-5001 USA

## Abstract

Engineering and evaluation of synthetic routes for generating valuable compounds require accurate and cost-effective *de novo* synthesis of genetic pathways. Here, we present an economical and streamlined *de novo* DNA synthesis approach for engineering a synthetic pathway with microchip-synthesized oligonucleotides (oligo). The process integrates entire oligo pool amplification, error-removal, and assembly of long DNA molecules. We utilized this method to construct a functional lycopene biosynthetic pathway (11.9 kb encoding 10 genes) in *Escherichia coli* using a highly error-prone microchip-synthesized oligo pool (479 oligos) without pre-purification, and the error-frequency was reduced from 14.25/kb to 0.53/kb. This low-equipment-dependent and cost-effective method can be widely applied for rapid synthesis of biosynthetic pathways in general molecular biology laboratories.

## Introduction

The development of high-throughput DNA sequencing and bioinformatics has generated massive amount of information about metabolic pathways of important compounds^1^. New genetic elements and pathways are required for understanding and engineering these synthetic pathways. Generally, *de novo* DNA synthesis is used for generating DNA sequences of genetic elements encoding members of pathways. The *de novo* synthesized DNA can be designed to specifically test hypotheses about the effect of the sequence on function, and for easy access of target sequences that are difficult to amplify or modify from natural sequences^2, 3^. Currently, the traditional approach that utilizes controlled-pore glass (CPG) oligos as starting materials for long gene assembly are in use. However, the high cost of CPG-oligos (~$0.10–0.20 per nucleotide) leads to an approximate price tag of $0.50 per base pair (bp) for gene synthesis^4–6^. Moreover, error removal in synthesized genes (2–5 error/kb)^3^ increases the production cost^7, 8^. Therefore, inexpensive microchip-derived oligos (Mcp-oligos) ($0.00001–0.001 per nucleotide) could be better as starting oligos for gene synthesis^6, 9–12^. However, inherently, Mcp-oligos consist of a low-concentration pool (10^4^–10^6^ molecules for each oligo)^10^ of thousands of oligos with various error rates (2–10/kb)^13^. The low concentration and high complexity of Mcp-oligos pose challenges for the effective assembly of long constructs. In addition, the high error-rate is difficult to rectify^9^, which is yet another limitation.

Various methods that use Mcp-oligos as building blocks for synthesizing long DNA exist^6, 10, 14^. Although these methods have circumvented majority of the technical problems such as low fidelity, low-yield, and high complexity of Mcp-oligo pools during gene synthesis, further modifications are still required for reducing cost, and for improving throughput and convenience to meet the needs of large-scale *de novo* DNA synthesis. *De novo* gene synthesis with Mcp-oligos includes 3 main steps: (i) parallel synthesis of designed oligos on microchip, and amplification and purification of Mcp-oligos; (ii) assembly of DNA by cycles of ligation or polymerization; (iii) error correction to improve the fidelity of the synthetic DNA. Selective oligo pool amplification^6, 8, 10, 15^ is the cornerstone of the strategy for obtaining enough oligos for subsequent DNA assembly. In this strategy, the large Mcp-oligo pools from one chip are divided into subsets (or subpools) using polymerase chain reaction (PCR)^6^ or isothermal oligonucleotide amplification^10^ with orthogonal primers in solution or on a solid surface. Each subpool contains only the oligos required for a unique fragment assembly. Although selective oligo pool amplification can simultaneously improve the amount of 10–20 oligos in one subpool^6, 9, 10^, the throughput is not high enough to perform large-scale DNA synthesis of several thousands of oligos or hundreds of subpools. The presence of low number of oligo sequences (10~20 oligos) in an assembly reaction improves the ease and effectiveness of DNA assembly^6^. However, large numbers of genes have to be assembled and synthesized for multiple gene synthesis, especially for constructing genetic circuits and entire genomes, and a strategy involving higher throughput assembly is a prerequisite in such cases. Currently, enzymatic mismatch cleavage (EMC) is the most popular method for removing errors^9, 13, 16, 17^. This multiple step approach includes fragment assembly, endo-/exo-nuclease digestion, and re-assembly of the fragments. MutS, the DNA mismatch-binding protein^15, 18, 19^ has also been used for removing errors in DNA. In this approach, MutS first recognizes and binds to the erroneous DNA, and the mismatched DNA-protein complex is removed by electrophoreses^19^, centrifugation^18^ or column separation^15^. However, this approach is applicable on only one fragment in each reaction, which therefore, increases the expense and time of the operation, and decreases the throughput of the method.

Currently, optimized and high-throughput synthetic methods for *de novo* DNA synthesis exist. Quan and co-authors developed an on-chip gene synthesis technology, which contains 30 physically separated cells on a microchip. In this method, all the oligos for a particular gene were synthesized using inkjet printing, amplified by isothermal amplification, assembled in one cell, and conducted 30 cells for parallel synthesis^10^. Mismatch-sensitive hybridization^9^ or high-throughput pyrosequencing-mediated error correction^8^ are also high-throughput approaches. However, instruments specialized for error-correction^8, 20^ and parallel operation, and next-generation sequencing (NGS) reaction platforms^9^ are required for effective large-scale synthesis^10^. The high-cost of these equipment and NGS increases the synthesis cost. Klein *et al*.^20^ reported multiplex pairwise assembly of oligos from 160-mer array-synthesized oligonucleotide pools to 2,271 targets of 192–252 bases using a ‘dial-out PCR’ protocol combined with Illumina sequencing for selecting error-free assembled DNA. However, assembly of two oligos limited the length of the target DNA (192–252 bp), and high-throughput sequencing increased the cost.

**Figure 1:**
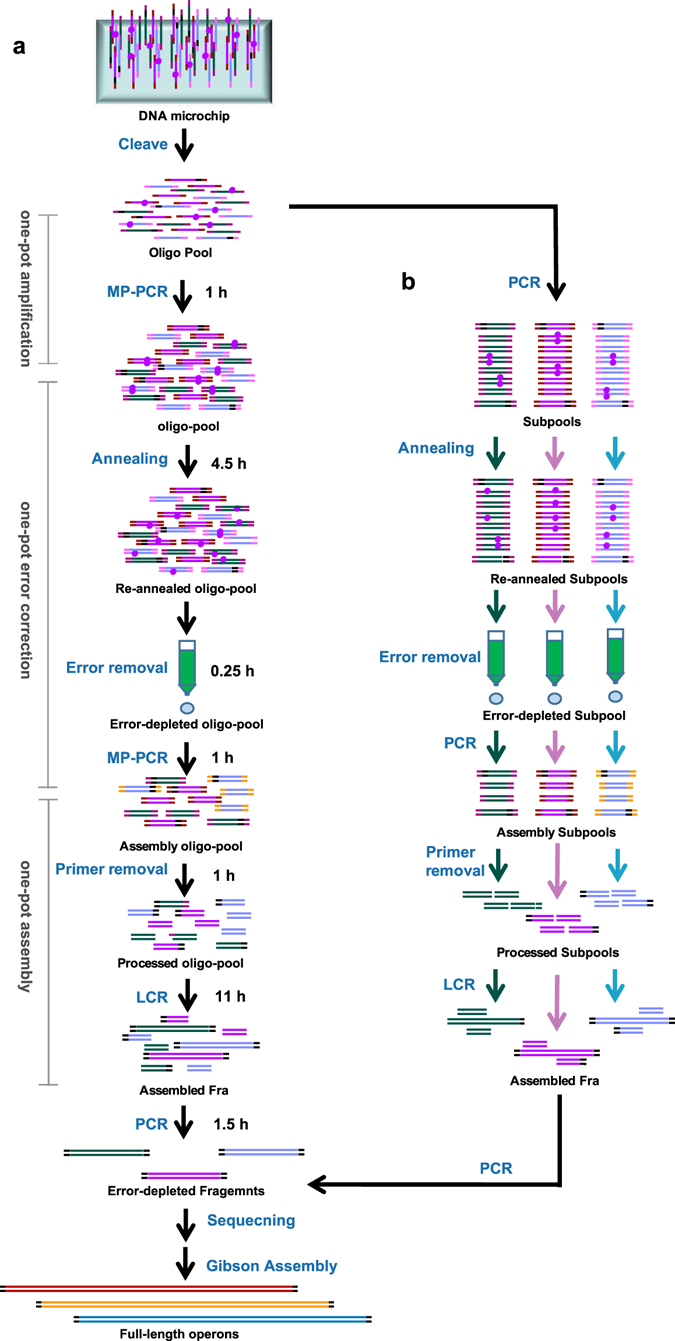
Overview of throughput-improved *de novo* gene synthesis. (**a**) Throughput-improved DNA synthesis strategy, in which the throughputs of oligo amplification, error-removal and assembly were improved to 479 unique oligos. (**b**) Low-throughput DNA synthesis strategy developed in our previous report.

In this study, we developed a low-equipment-dependent process, in which the throughput of each synthetic reaction (oligo amplification, error-removal, and DNA assembly) was highly improved using the commercial Mcp-oligo pools. The advantages of this method are its low operation cost, and labour savings on parallel operations and reaction platforms. As shown in Fig. 1, the process includes three steps: i) amplification of the entire Mcp-oligo pool template cleaved from the microchip using multiplex PCR (MP-PCR). ii) high-throughput error correction, including re-annealing of the entire amplified oligo pool to expose the erroneous nucleotides as mismatches, removal error of entire oligo pool using MutS-immobilized cellulose column (MICC), and MP-PCR to amplify the entire error-depleted oligo pool from the collected eluates. iii) multiplex assembly. After removing the primer sequences of the oligos by type IIS restriction endonucleases (*Mly* I), the obtained double-stranded DNAs were assembled to target fragments via ligase chain reaction (LCR). A second round of the error-correction procedure for further reducing error rates followed this step, if required. The target error-free fragments were verified by sequencing. The error-free fragments can be used for the construction of metabolic pathway operons and characterization of the constructs.

## Results

### Oligo design of lycopene biosynthetic pathway

**Figure 2:**
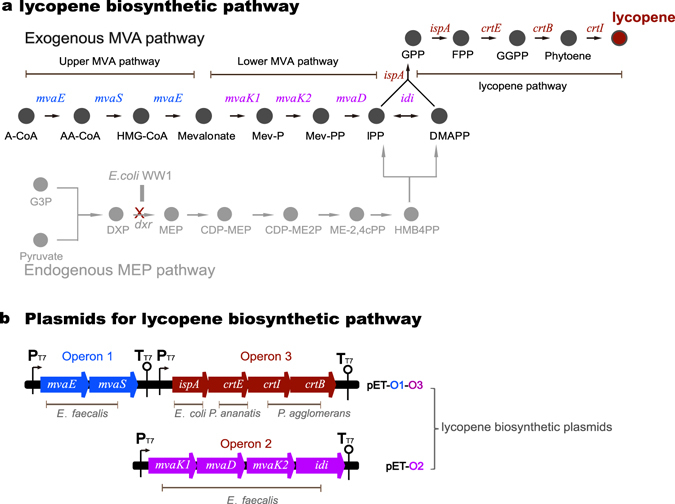
The heterogenous lycopene biosynthetic pathway constructed in this study. (**a**) Depiction of the synthetic pathway. The endogenous MEP pathway of *E. coli* is indicated in gray. The lycopene biosynthetic pathway, including the MVA and lycopene pathways is depicted in black. (**b**) Plasmids and indicated operons for lycopene production (information about genes is provided in the Supplementary Data). *mvaE*, hydroxymethylglutaryl-CoA reductase; *mvaS*, hydroxymethylglutaryl-CoA synthase; *mvaK1*, mevalonate kinase; *mvaK2*, phosphomevalonate kinase; *mvaD*, diphosphomevalonate decarboxylase; *idi*, isopentenyl-diphosphate delta-isomerase; *ispA*, farnesyl diphosphate synthase; *crtE*, geranylgeranyl pyrophosphate synthase; *crtB*, phytoene synthase; *crtI*, phytoene dehydrogenase; A-CoA, acetyl-CoA; AA-CoA, acetoacetyl-CoA; Mev-P, mevalonate 5-phosphate; Mev-PP, mevalonate pyrophosphate. HMG-CoA, hydroxymethylglutaryl-CoA; IPP, isopentenyl diphosphate; DMAPP, dimethylallyl diphosphate; GPP, dimethyl pyrophosphate; FPP, farnesyl diphosphate; GGPP, geranylgeranyl pyrophosphate; G3P, glyceraldehyde 3-phosphate; DXP, 1-deoxy-D-xylulose 5-phosphate; dxr, DXP reductoisomerase; MEP, 2-C-methyl-D-erythritol 4-phosphate; CDP-ME, 4-diphosphocytidyl-2-C-methyl-D-erythritol; CDP-ME2P, 4-diphosphocytidyl-2-C-methyl-D-erythritol 2-phosphate; ME-2,4cPP, 2-C-methyl-D-erythritol 2,4-cyclopyrophosphate; HMB4PP, 1-hydroxy-2-methyl-2-(E)-butenyl 4-pyrophosphate.

We used the *de novo* synthesis of lycopene biosynthesis pathway for evaluating the performance of the throughput-improved process involving Mcp-oligos for DNA synthesis in this study. Ten codon-optimized genes (891–2412 bp, 11.8 kb in total), including the genes for heterologous lycopene production (*crtE*, *crtB*, crt*I*, and *ispA*) and mevalonic acid (MVA) pathway (*mvaE*, *mvaS*, *mvaK1*, *mvaK2*, *mvaD*, and *idi*) (for enhancing the supply of precursor molecule IPP for lycopene production^21^) (Fig. 2a) were synthesized and expressed in *Escherichia coli*. The lycopene biosynthesis pathway was selected because lycopene is a valuable carotenoid widely used in pharmaceutical and nutraceutical industries^22, 23^. In addition, it is easy to evaluate lycopene production via the detection of red color in *E. coli* cells^24^.

Oligos for synthesizing the genes of the lycopene biosynthetic pathway were designed by DNAworks^25^. The gene sequences were split into overlapping fragments using DNAWorks and flanked by common primer pairs (fragment-specific primers: Fra-F and Fra-R in Supplementary Table S1) for convenient analysis and amplification. Then, the ribosome-binding site (RBS) sequences were flanked by the ends of the contiguous genes. Using the overlaps, the fragments and RBSs could later be assembled into operons by the Gibson assembly method^26^. These fragments were further split into overlapping oligos with similar melting temperatures and with no gaps between contiguous overlaps (see Supplementary Fig. S1) using DNAworks such that they could later be assembled by LCR. Each oligo from a fragment was flanked with orthogonal primer pairs (oligo-specific primers) for oligo amplification (see Supplementary Tables S1 and S2). All designed oligos of these ten genes formed one oligo pool (lycopene pool), containing 479 oligos of different lengths (64–124-mer), on a photo-programmable microfluidic microchip^12^. The long Mcp-oligos (100–200 mer) from Agilent technologies (Santa Clara, CA, USA) are not commercially available, and the Mcp-oligos from Twist Bioscience (San Francisco, CA, USA) are not convenient for use. Therefore, we used the commercial Mcp-oligos from LC Science (Houston, USA), although the long oligos showed a relatively higher error rate (without error correction, only 3.23% assembled fragments were correct and the error rate was 14.25/kb (Table 1).

**Table 1:** Error analysis of assembled fragment sequences using different error-removal strategies with various throughputs

Error type	Non-error removal	Low-throughput error removal	Throughput-improved error removal	Multiplex assembly	Double round error removal^a^
Multi-error^b^	6	5	0	2	0
Deletion	175	7	4	4	1
Insertion	38	10	9	11	0
Substitution	229	27	23	29	4
Total errors	448	49	36	46	5
Bases sequenced	31445	23599	16343	24874	9243
Error frequency (error per kb)	14.25	2.08	2.20	1.85	0.54
Ratio of error-free synthetic DNA^c^ (%)	3.23	48.44	41.51	55.5	81.48

^a^Error-removal was conducted both at oligo and fragment stages.

^b^Error site located on sequence that contains more than three adjacent consecutive nucleotide errors.

^c^Length of synthetic DNA was 319–423 bp for lycopene biosynthesis genes (without primer sequences).

### Low-throughput DNA synthesis strategy was an effective control

We used the the low-throughput DNA synthesis process, in which the throughput of each step was one subpool (Fig. 1b), as a control in our study. In brief, each subpool was selectively amplified from the Mcp-oligo pool, processed for error-removal using MICC, and assembled using LCR. The error-correction process by MICC includes three steps: i) re-annealing of DNA to expose the erroneous nucleotides as mismatches, ii) remove of these mismatches by the MICC, and iii) re-amplification of error-depleted oligos from the recovered eluates. The assembled fragments were sequenced thereafter to determine the error rates.

**Figure 3:**
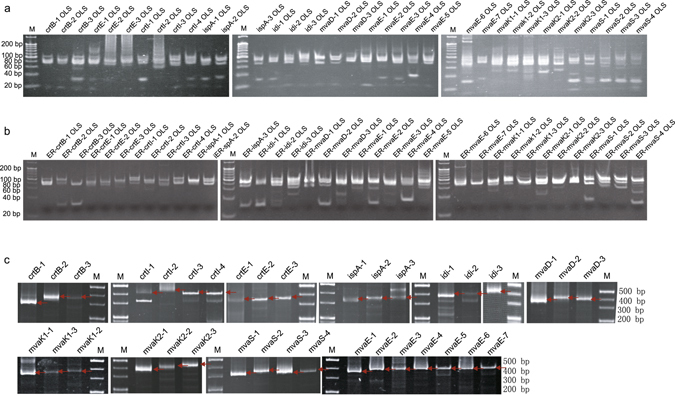
Low-throughput synthesis of gene fragments. (**a**) Selective amplification of each subpool for generation of lycopene biosynthetic genes. Subpools were directly amplified using the oligo pool cleaved from the microchip as the template. (**b**) PCR of each error-depleted subpool. The oligo subpool was amplified with the error-depleted elute from etMICC as the template. (**c**) Fragments assembled by LCR from error-depleted oligo subpools. M, 20 bp DNA ladder (TaKaRa Bio). Red arrows indicate target fragments. OLS, oligo subpool; ER, error-removed.

Each oligo subpool was balance-amplified from the oligo pool or error-depleted subpool and assembled into the target fragment using the low throughput strategy. As shown in Fig. 3a and b, all the 36 subpools were selectively amplified from the Mcp-oligo pool or from the eluate of the error-depleted oligo pool, and all the fragments were successfully assembled from the error-depleted oligo subpools (Fig. 3c). The oligo subpools without error-removal were also assembled into fragments as a control.

The fidelity of the synthetic fragments generated using the low throughput synthesis strategy was dramatically improved. In the absence of error removal, the fidelity of the synthetic fragments using Mcp-oligos was poor, with an error rate of 14.52/kb (448 errors in 31445 bp) and only 3.23% fragments were error-free (Table 1). However, as shown in Table 1, the error rate of the fragments assembled from the error-removed oligos was reduced 6.58-fold (from 14.52 /kb to 2.08/kb), and the error-free fragment ratio improved 15-fold (3.23% to 48.44%). These results indicated that error removal using a MICC dramatically improved the fidelity of the synthesized genes, which was consistent with our previous report^15^.

### A relatively high-throughput DNA synthesis approach with entire oligo amplification, error removal, and fragment assembly was established

We included a series of throughput-improved reactions to develop a relatively high-throughput approach for *de novo* gene synthesis.

**Figure 4:**
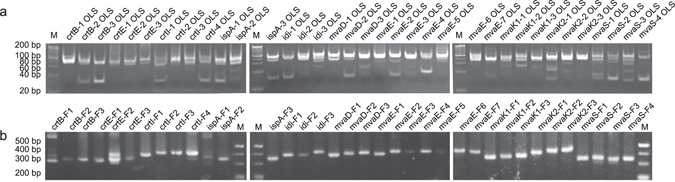
Assembly of fragments using re-amplified subpools from MP-PCR amplicons. (**a**) Amplification of oligo subpools from the MP-PCR amplicons prior to throughput-improved error correction. (**b**) Each fragment was assembled by LCR using re-amplified subpools from MP-PCR amplicons for evaluating the oligo quality of MP-PCR product. M, 20 bp DNA ladder (TaKaRa Bio); OLS, oligo subpool; F, fragment.

First, MP-PCR-based oligo amplification was optimized to amplify the entire oligo pool (Fig. 1). We adopted the subpool separation strategy during the oligos designing for improving the flexibility of DNA assembly^6, 8, 10^. MP-PCR, which can amplify several sequences simultaneously in the same reaction using multiple primer pairs, has been widely used in nucleic acid diagnostics^27, 28^, and was utilized for oligo pool amplification. In this study, 36 subpools (479 oligos) were amplified in one MP-PCR reaction with orthogonal primers. To evaluate the suitability of the amplified product quality for subsequent DNA assembly, the products were processed using the low-throughput re-amplification, error correction, and assembly steps. All the 36 target subpools were separately re-amplified from the MP-PCR amplicons (Fig. 4a) and were assembled into target fragments (Fig. 4b). These results showed that 36 subpools (479 oligos) could be amplified simultaneously in one MP-PCR reaction without missing target oligos, and that the concentration of each oligo was sufficient for fragment assembly.

**Figure 5:**
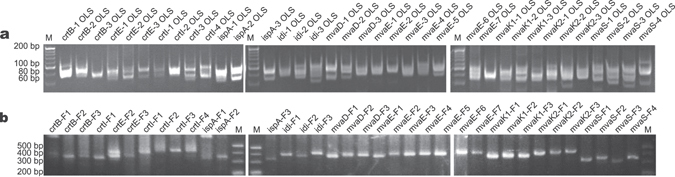
Assembly of fragments using oligos after entire pool annealing and error-removal by MICC. (**a**) Amplification of oligo subpools from the MP-PCR amplicons after throughput-improved error correction. (**b**) Each fragment was assembled by LCR from error-depleted oligo subpools. M, 20 bp DNA ladder (TaKaRa Bio); OLS, oligo subpool; F, fragment.

A highly efficient throughput-improved error-correction method was constructed after optimization of the entire oligo amplification approach. The error correction approach included re-annealing, error removal, and re-amplification steps. Re-annealing is the first step that links amplification to error-removal. In the throughput-improved re-annealing process, all oligos (36 subpools, 479 oligos) in the MP-PCR amplified oligo pools (MP-Pools), which possessed more complex composition than that of a single subpool, were re-annealed to expose synthesis errors. In the error removal step, the oligo pool containing 36 re-annealed subpools was loaded onto one etMICC. The error-depleted oligos were collected, selectively amplified, assembled, and analysed. PCR selectively amplified all the 36 subpools from the eluted DNA fractions, which were assembled into target fragments using LCR (Fig. 5).

The high fidelity of DNA synthesis was retained using amplification and error removal of the entire oligo pool. The fidelity of the assembled fragments improved 6.48-fold (from 14.25/kb to 2.20/kb), and the ratio of error-free sequences improved 12.85-fold (from 3.23% to 41.51% (Table 1). All fragments were assembled, and the error rate was similar to that of the low-throughput approach when up to 36 subpools (479 oligos) were used in the throughput-improved error-correction step (Table 1). Therefore, we successfully established a highly efficient error-correction method combined with amplification of the entire oligo pool.

**Figure 6:**
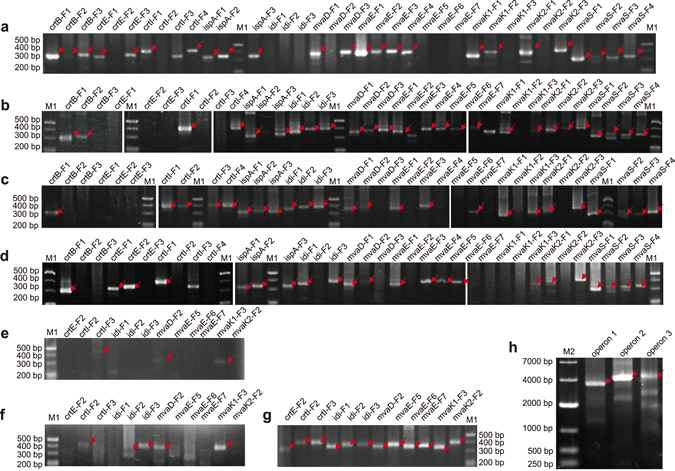
*De novo* synthesis of lycopene biosynthetic pathway. Fragments were assembled by multiplex assembly using error-depleted oligo pools of various throughputs and half-specific primer pairs. Thirty six (**a**), 3–4 (**b**), 6–7 (**c**), and 17–19 (**d**) subpools were used in one assembly reaction and 24, 26, 24, and 22 fragments were assembled, respectively. (**e**) Fragment detection in the assembly (36 subpool/reaction) with full-specific primer pairs. (**f**) Fragment detection in the assembly (12 subpool/reaction) with half-specific primer pairs. (**g**) Fragment detection in the assembly (12 subpool/reaction) with full-specific primer pairs. (**h**) Operons assembled by Gibson assembly. M1, 20 bp DNA ladder (TaKaRa Bio); M2, 2 K plus II DNA ladder (TaKaRa Bio). Red arrows indicate target fragments. OLS, oligo subpool; F, fragment.

Subsequently, we optimized and evaluated a multiplex fragment assembly method. First, the error-removed MP-pool containing the 36 error-depleted subpools was used to assemble the 36 target fragments in one LCR. After assembly, each target fragment was amplified with one specific fragment primer and one F-primer (half specific primer pair) (see Supplementary Tables S1 and S2). However, only 66.7% (24/36) of the target fragments were obtained (Fig. 6a). To investigate whether the failure in the assembly of all target fragments was due to the presence of excessive subpools or oligos in the assembly reaction, lesser amounts of subpools (3–4, 6–7 or 17–19 subpools) in one assembly reaction were used. The results were similar to those obtained with 36 subpools, and approximately 66% fragments were assembled, although the successfully assembled fragments were different (Fig. 6b–d). Furthermore, to understand whether the failure in the assembly of the 12 target fragments in one assembly reaction was due to ineffective amplification by the half-specific primer pairs, two specific fragment primers (full-specific prime pairs) (see Supplementary Tables S1 and S2) of each fragment were used. Consequently, three of the 12 fragments were further detected (Fig. 6e). We concluded that both PCR efficiency and the complexity of the oligo pools contributed to the failure in fragment assembly. Based on above results, we attempted to assemble these 12 fragments in another multiplex assembly reaction. MP-pool containing the 12 error-depleted oligo subpools corresponding to the 12 fragments was assembled in one reaction. Only 5 of the 12 were detected using half-specific primer pairs for fragment amplification (Fig. 6f). However, all the 12 fragments were detected when full-specific primer pairs were used for fragment amplification (Fig. 6g). Therefore, all 36 target fragments could be assembled using two rounds of multiplex assembly and full-specific primer pairs.

There was no obvious reduction in the fidelity of the synthesized fragments with the high-throughput DNA synthetic strategy. The fidelity of the assembled fragments was determined by sequencing. As shown in Table 1, the fidelity of the assembled fragments obtained using multiplex assembly improved 7.70-fold (error frequency was reduced from 14.25/kb to 1.85/kb). Moreover, the ratio of error-free sequences improved 17.18-fold (from 3.23% to 55.50%) (Table 1). These results indicated that the multiplex assembly procedure was not error-prone.

### Error-removal of assembled fragment pool could further improve the fidelity of the assembled fragments

Iterated error removal at fragmentation stage could further reduce errors pertaining to DNA synthesis. Errors were removed by etMICC after all 36 fragments were re-annealed, and the error rate of the fragments was further reduced to 0.54/kb from 1.85/kb (Table 1). Therefore, with two rounds of error-removal during *de novo* gene synthesis, the error rate of the synthetic fragments was reduced by a factor of at least 26 and > 80% of the fragments were error-free (Table 1).

### The lycopene biosynthetic pathway was functional

To evaluate the function of the lycopene synthetic pathway constructed using the synthetic error-free fragments, three long operon DNAs were assembled using Gibson assembly method^26^ with the synthetic error-free fragments and RBS sequences. As described in Fig. 6h, the three full-length operons were successfully assembled. Then, the assembled operons were inserted into expression vectors to generate plasmids pET-O1-O3 and pET-O2 (Fig. 2). We also evaluated the traditional method of overlap-extension PCR that assembles each fragment into full-length genes and constructs operons. Both methods successfully generated the operons. However, the full-length DNA assembly could be integrated with operon construction with higher efficiency using the Gibson assembly method.

Furthermore, the function of the synthesized lycopene biosynthetic pathway was evaluated in a strain harboring a block in the 2-C-methyl-D-erythritol 4-phosphate (MEP) pathway. The *E. coli* stain WW-1 with *dxr* deletion harboring the heterogenous lycopene biosynthetic pathway (including lycopene pathway and MVA pathway) turned red due to the accumulation of lycopene (see Supplementary Figs S2 and S3) (the construction of this stain WW1 is described in Supplementary methods).

## Discussion

The throughput of oligo amplification was highly improved by MP-PCR. Subpool separation using PCR amplification is a common strategy^6, 15^ for gene synthesis using Mcp-oligos. However, the throughput of amplification was low ( < 32 oligos/reaction)^15^ in the single subpool separation strategy, which limited the scalability of gene synthesis involving hundreds of subpools. Increasing the number of subpools for amplifying more target genes in one PCR reaction would reduce operation cost and time. Therefore, we first attempted to amplify all oligos (391 unique oligos) for 21 different genes encoding the red fluorescent protein with one pair of primers. However, high-throughput sequencing (GAIIx sequencing, Illmina) detected severe imbalance of oligos, although all the oligos were detected. Three hundred and seventy-eight oligos were detected in the recovered sample after error-removal using MICC, which was 96.68% of all oligos. Approximately three percent oligos of the oligo pool was lost during the process, and the imbalance of oligos was evident. Therefore, we used a combination of MP-PCR for amplifying oligos and subpool separation to circumvent these problems. The amplification throughput was improved from 1 subpool to 36 subpools (479 oligos) in one reaction, and all 36 fragments were successfully assembled into the target fragments with these amplified oligos (Fig. 4). Therefore, all oligos were amplified and the concentration of each oligo was enough for successful target fragment assembly even without high-throughput sequencing. These results indicated that MP-PCR is helpful to avoid the extreme bias during oligo pool amplification.

Throughput of error-correction of oligos improved to 479 oligos per MICC in the throughput-improved approach. All synthesized oligos released from the microchip formed a highly complex pool, which contained oligos for target genes, truncated oligos, and oligos with various errors. In general, the error rate is 6–25 /kb for Mcp-oligos without special pretreatment^9, 17, 29^. Thus, error-removal is an unavoidable step in most *de novo* gene synthesis process using Mcp-oligos for obtaining DNA with an error rate lower than 1 per kb. In a previous study regarding *de novo* gene synthesis with Mcp-oligos, error-removal was a time and cost inefficient step^30^. Several methods, including high performance liquid chromatography (HPLC) and polyacrylamide gel electrophoresis (PAGE)^9^, mismatch-sensitive hybridization^9^, EMC^6, 10, 16^, DNA mismatch binding proteins^18, 19^ and next-generation sequencing (NGS) platform-based^8, 20, 31^ error-correction methods have been reported to improve the fidelity of the synthetic DNA before and after assembly. The throughput of error-corrected methods via EMC and DNA mismatch binding proteins are generally low (1 fragment/reaction, or 10–32 oligo/reaction^15, 16^). The throughput of error-corrected methods via HPLC and PAGE, mismatch-sensitive hybridization, and next-generation sequencing could be high (>10^3^ oligo/reaction). However, the HPLC and PAGE methods are limited to oligos with equal length, and the error-corrected efficiency is low (<3-fold improvement)^9^. Therefore, improved and superior methods are required. Mismatch-sensitive hybridization requires two micro-chips and specific hybridization equipment; however, the improvement in fidelity was only ~8-fold. The efficiency of next-generation pyrosequencing platform-mediated error-correction methods is very high (~500-fold improvement). However, these methods require highly specialized equipment, and not all methods are compatible for use with NGS platforms. Schwartz *et al*.^31^ reported another NGS-based method that is compatible to any NGS-sequencer, but it significantly increased the total gene synthesis time. In addition, these NGS-based methods require using the tedious dial-out PCR for the selective amplification of error-free DNA^31^, and the synthesis of large number of dial-out primers increases the operation cost. MICC has previously been shown to efficiently generate high-quality DNA from Mcp-oligos at extremely low cost^15^. However, a previous report showed that the error removal throughput of MICC was not high (10–32 oligos/MICC), which may limit the scalability of DNA synthesis. Therefore, the throughput has to be improved further to achieve error-free megabase-scale assemblies. Fortunately, PCR amplification of oligo subpools facilitated error correction with small amounts of error-depleted oligos that could be amplified to meet the requirements of gene assembly. Expansion of the scale of this MICC method for high-throughput error-removal was possible. Although the throughput of each MICC improved from 10 oligos to 479 oligos in this study (1 to 36 subpools/MICC), the errors in amplified oligos could be removed with similar efficiency (Table 1).

Our approach also improved the throughput for oligo pool assembly. The throughput of Mcp-oligos in a DNA assembly reaction via LCR was typically not more than 32 oligo/reaction (not more than 3 target fragments/reaction)^15^. In this study, the entire oligo pool was used to assemble to dozens of fragments in one reaction. All the 36 fragments were successfully assembled from the error-depleted Mcp-oligos pool in the throughput-improved mode (Table 1) with 2 cycles of multiplex assembly reaction, and the obtained fragments were not identical in each round. Recently, with the development of DNA array-synthesis, Customarray is synthesizing high quality Mcp-oligo pools with longer length (<200-mer). In 2016, Klein *et al*. reported a protocol for high-throughput assembly of Mcp-oligo pool containing ~4,000 oligos (160-mer) representing 2,271 targets of 192–252 bases. Though the throughput was significantly improved, assembly with only 2 oligos limited the length of the target DNA (192–252 bp). In addition, this method has to be combined with an NGS error-correction system and the labour-intensive and expensive dial-out PCR. However, a combination of their strategy and our approach would facilitate the assembly of larger numbers and longer DNA using Mcp-oligo pool in future.

The unsuccessful assembly in the multiplex assembly approach occurred due to the non-uniformity of oligos in the oligo pool. We obtained only 24 out of 36 fragments when we attempted to assemble 36 fragments in one reaction, and another round of assembly was required for assembling the remaining 12 fragments. Nonetheless, certain fragments failed to assemble even in the assembly reaction with less subpools per reaction (3–4, 6–7 or 17–19 subpools) (Fig. 6b–d). In addition, the successfully assembled fragments were different in each approach (Fig. 6b–d). These results indicated that even though the oligos in each subpool were complete, the non-uniform distribution of each oligo in the error-depleted pools might cause unsuccessful assembly of certain target fragments. Furthermore, the secondary structure of oligos may also increase the difficulty of various fragment assemblies.

Oligo synthesis, amplification, and error correction may result in the non-uniform distribution of each oligo in the assembly pool, although the imbalance is not severe in each step. However, the bias can accumulate and cause failed fragment assembly. The synthesis efficiency of each oligo during the array synthesis process is different and may result in variations in the quality and quantity of each oligo. Although MP-PCR could be helpful to alleviate the bias during amplification, the varied amplification efficiency of each primer pair and abundance of the synthesized oligos (template) could still introduce bias in amplification. In addition to error correction, annealing of oligos with various abundances can also decrease the uniformity of oligos.

Further optimization of sequence-design algorithms for oligo design with considerations for sequence length, GC content, structure, melting temperature, and reaction condition are required. Sequence-optimization algorithms have been used to eliminate sequence features that interfere with DNA assembly^32^. Certain optimization software such as DNAWorks^25^ and TmPrime^33^, which have been built for oligo designing, can reduce the difficulties encountered during the assembly process. However, to avoid interference of oligos from the same pool for multiple fragments, algorithms that can optimize oligo design for large-scale DNA synthesis with Mcp-oligos are required. Careful design, and selection and coordination of primer pairs for different subpools are also necessary for achieving balanced amplification of each oligo in each MP-PCR. Furthermore, each primer pair can still be used independently in MP-PCR if certain fragments fail to assemble in the throughput-improved strategy. Such fragments can be assembled using the single subpool strategy.

The synthesis scale of our approach can be further improved in combination with existing technologies. A previous report showed that 836 subpools from an oligo library synthesis pool containing ~13,000 oligonucleotides could be amplified with well-designed ‘orthogonal’ primers^6^. The combination our method with the method described above could improve the scale significantly through parallel operations. Although amplification of oligos and error removal over 500 were not evaluated, the hundreds of subpools could be divided into several larger subpools containing ten normal subpools (oligos for one fragment in one subpools). In this study, we synthesized ~12 kb fragments using the throughput-improved protocol. Therefore, for synthesizing 100 kb, approximately 8 sets of parallel experiments are required, which can be completed by one person in 2–3 days. Finally, our method includes the option of partially roboticization, and the scale for DNA synthesis can be easily modified to generate billions of bases if instruments for parallel operation such as general automatic plate coating machine are available.

The simplified procedure and throughput-improved strategy of *de novo* gene synthesis using Mcp-oligos, significantly reduced the cost of *de novo* gene synthesis. First, compared to the previous MICC low throughput strategy^15^, which included entire pool amplification and error removal of the oligo pool, the cost of oligo amplification and error-removal was reduced 36-fold. Second, multiplex assembly of fragments is superior to the traditional approach that necessitates one assembly reaction per fragment. The cost of fragment assembly was reduced more than 18-folds (36/2) using our approach. In addition, less time is required for *de novo* gene synthesis with the improved throughput approach.

In addition to than the relatively low cost of materials and labour, and reduced operation time, this method also eliminated the economic burden of using special equipment for large-scale DNA synthesis. A set of techniques have recently been developed for *de novo* gene synthesis on large-scale, which allows assembly of gene-sized DNA using Mcp-oligos^3, 8, 10^ at low cost. However, special equipment is required for such large-scale synthesis, which increases cost apportionment that may not be available in a general laboratory. However, the method described in this study utilizes general molecular biological equipment such as thermocycler and commercially available Mcp-oligos. Therefore, this method could be useful in specific applications involving high-throughput DNA synthesis.

The method described in this study enables precise *de novo* synthesis of DNA cassettes using Mcp-oligos, which can be assembled into larger constructs like pathways or gene clusters with general molecular biology instruments. This method has the potential of significantly reducing the cost associated with the generation of high-quality synthetic DNA. Moreover, the procedure for throughput-improved long DNA synthesis can be scaled up for high-throughput error-free gene synthesis.

## Methods

### Chemicals and strains

All chemicals were of reagent-grade or higher and were purchased from Sangon Biotech Co. (Shanghai, China) unless otherwise noted. All restriction enzymes and T4 DNA ligases were obtained from Thermo Fisher Scientific Inc. (Waltham, MA, USA). PrimeSTAR HS DNA polymerase was purchased from TaKaRa Bio (Dalian, China). KOD Plus DNA polymerase was obtained from TOYOBO (Osaka, Japan). Taq DNA ligase was purchased from New England Biolabs (Beverly, MA, USA).

### Synthesis of oligo pool on microchip

Oligos were synthesized on a photo-programmable 4k microfluidic microchip^9, 12^, with 7 points for one oligo. After synthesis, the oligos were cleaved by ammonium hydroxide, followed by vacuum evaporation for removing excess ammonium hydroxide and for concentrating the oligos. The oligo pool, containing 20 pmol of oligos without additional purification^9^, was used as the template for oligo amplification in the following experiments.

### Oligo amplification

The oligos were amplified using PCR with orthogonal primer pairs through selective amplification and MP-PCR amplification. Unlike selective PCR amplification, in which one subpool is amplified per reaction, several or all subpools were amplified in one reaction with multiplex orthogonal primer pairs in MP-PCR, which were mixed in equal amounts as primers. PCR reactions were performed using KOD plus DNA polymerase as described in Supplementary Methods.

### MutS-immobilized cellulose column preparation

etMICC was constructed as described previously^15^. Briefly, 600 pmol each of eMutS and tMutS were mixed with 500 µl of the RAC slurry (20 mg/ml) and incubated at room temperature for 10 min. Then, the mixture was loaded onto an empty chromatography column. After the slurry settled in the column, 1 ml of binding buffer (5 mM MgCl2, 100 mM KCl, 20 mM Tris-HCl (pH 7.6) and 1mMDTT) were added to wash away the free proteins.

### Error-removal of oligos and fragments using etMICC

For error removal, oligos were re-annealed and loaded onto one etMICC as described previously^15^. After the eluted fractions containing the error-depleted subpools were recovered, another round of oligo amplification was performed to obtain each unique error-depleted subpool or MP-pools.

For error removal of fragments, the total fragment mixture (3 µg) of all 36 re-annealed fragments was mixed in equal amounts and loaded onto the etMICC. The first eluted fraction containing the error-depleted fragments was recovered. Subsequently, the eluate was used as a template to separately amplify each fragment using Pfu DNA polymerase as described in Supplementary Methods.

### Fragment assembly by LCR

For fragment assembly, the primer regions of the oligos were removed using *Mly*I digestion^15^. The 10 µl LCR was performed in reaction mixtures containing 1 µl 10 × Taq DNA ligase buffer, 10 pmol (~500 ng) of primer-removed oligos and 1 µl Taq DNA ligase. Cycling parameters were 95 °C for 5 min, followed by 30 cycles of 30 s at 94 °C, and 20 min at 50 °C. After ligation, the assembled target fragments were amplified by PCR using the KOD plus DNA polymerase as described in Supplementary Methods.

### Sequencing of synthetic lycopene fragments

Error rates of the *de novo* synthesized fragments were evaluated via Sanger sequencing of random selected clones and analysed as reported previously^15^.

### Gibson assembly of lycopene biosynthetic operons

For Gibson assembly of each operon, the T7 promoter, the ribosome-binding site (RBS) of pET-21c, synthetic error-free fragments of *mvaE* and *mvaS* and the dsDNA of RBS 1 (see Supplementary Table S1) of *mvaS*, constituting operon 1, were mixed in equal amounts. Synthetic error-free fragments of *mvak1*, *mvaD*, *mvak2*, and *idi*, and the dsDNA of RBS 2, 3, 4 of *mvaD*, *mvak2*, and *idi*, respectively, constituting operon 2, were mixed in equal amounts. The T7 terminator of pET-21c, synthetic error-free fragments of *ispA*, *crtE*, *crtB*, and *crtI* and the dsDNA of RBSs 5, 6, 7 (see Supplementary Table S1) of *crtE*, *crtB*, and *crtI*, respectively, constituting operon 3, were mixed in equal amounts and used as template for the Gibson assembly reaction. The detailed information for the Gibson reaction is described in Supplementary Methods. After assembly, full-length operons 1, 2, and 3 were amplified separately with primers T7F-H-F and mS-X-R, mk1-N-F and ii-H-R, iA-N-F and T7T-H-R, respectively (see Supplementary Table S1), using PrimeSTAR HS DNA polymerase as described in Supplementary Methods.

### Construction of expression plasmids of lycopene biosynthetic pathway

Operon 1 was inserted into the *Hind*III-*Xho*I sites of pET-21c to generate the plasmid pO1. Subsequently, operon 3 was inserted into pO1 at *Nhe* I-*Hind*III sites to generate the plasmid pO1-O3 (Fig. 2). Operon 2 was inserted into the *Nhe*I-*Xho*I sites of pET-28a to generate the plasmid pO2 (Fig. 2).

### Production of lycopene through synthetic pathway

The WW1 strain was cultivated in Luria-Bertani (LB) medium (containing 100 µg/ml ampicillin, 50 µg/ml kanamycin, and 2% glycerol) and induced by isopropyl-β-D-thiogalactopyranoside (IPTG, final concentration 0.1 mM) when the OD_600_ was 0.6 at 30 °C for 24 h. The JM109 (DE3) strain harboring pET-21c and pET-28a was used as a control. Red cell indicated lycopene production.

## Electronic supplementary material

Supplementary information
