# Supplementary material for: High-fidelity de novo synthesis of pathways using microchip-synthesized oligonucleotides and general molecular biology equipment
Source: Sci Rep. 2017 Jul 21;7:6119. doi: 10.1038/s41598-017-06428-0 (PMC5522410; doi:10.1038/s41598-017-06428-0)
Supplement: Supplementary file 1 — Supplementary information [file 41598_2017_6428_MOESM1_ESM.pdf]

Supporting information

High-fidelity *de novo* synthesis of pathways using microchip-synthesized oligonucleotides and general molecular biology equipment

Wan Wen[2], Lu Min[1], Wang Dongmei[1], Gao Xiaolian[1,3] , Hong Jiong[1]*

[1]School of Life Sciences, University of Science and Technology of China, Hefei, Anhui 230027, China

[2]The Key Laboratory of Biotechnology for Medicinal Plant of Jiangsu Province, Jiangsu Normal University, Xuzhou, Jiangsu, 221116, China

[3]Department of Biology and Biochemistry, University of Houston, Houston, TX77004-5001, USA

Correspondence: hjiong@ustc.edu.cn

	Name	Page
Supplementary Table S1	Primers and RBS sequences used in this study	3-5
Supplementary Table S2	Primers used in lycopene gene synthesis	6
Supplementary Table S3	Summary of oligonucleotides design of lycopene biosynthesis genes	7
Supplementary Table S4	Primers used in the construction of strain WW1	8
Supplementary Figure S1	Design of oligonucleotides of lycopene pathway genes for synthesis on microchips (*mvaS* oligonucleotides are described as an example).	9
Supplementary Figure S2	Schematic representation of the construction of the WW1 strain harboring the genes of the lycopene biosynthetic pathway	10
Supplementary Figure S3	Production of lycopene via the engineered lycopene biosynthesis pathway	11
Supplementary Methods	Construction of *dxr*-deleted *Escherichia coli* WW1 and expression of the synthetic lycopene pathway	12
Supplementary Methods	PCR amplification of oligos	13
Supplementary Methods	PCR amplification of error-depleted fragments	13
Supplementary Methods	Gibson assemble of operons	14
Supplementary Methods	PCR amplification of full-length operons	14
Supplementary data		15-48
Supplementary Reference		49

Supplementary Tables:

Supplementary Table S1 Primers and RBS sequences used in this study.

Primers ID	Sequence[a]
MlyI_1F	CACAGGAGTCCTCAC
MlyI_2F	CCAAGGAGTCGCTAG
MlyI-3F	CAGAGGAGTCCTGAG
MlyI-4F	CCTACGAGTCGCAAC
MlyI-5F	CATTCGAGTCGTCTG
MlyI-6F	CGTACGAGTCCCTTC
MlyI-7F	CTGTCGAGTCTGTAT
MlyI-8F	GGTACGAGTCCGATC
MlyI-9F	CTTACGAGTCGTCTC
MlyI-10F	GGATCGAGTCTCTAC
MlyI-11F	GTGTGGAGTCATATG
MlyI-12F	GGATCGAGTCAATTC
MlyI-13F	CGTTCGAGTCATCCA
MlyI-14F	GCATGGAGTCGGATG
xMlyI_1F	GTATAGAGTCAGTCG
xMlyI-2F	GTCACGAGTCATGGC
xMlyI-3F	GATAAGAGTCTCAGC
xMlyI-4F	GAGTGGAGTCTACCG
Fra-F	ACGCTCTGAAGACCC
Fra-R	CGAGATAGAAGACAG
mvaE-F	ATGAAAACCGTTGTTATCATCGACG
mvaE-2-R	TTGAAAACGGTTTTCAGGGT
mvaE-3-F	GGGCACCCTGAAAACCGTTT
mvaE-4-R	CAGATAGTCGGTTTCGTCA
mvaE-5-F	GACCGTTGACGAAACCGAC
mvaE-5-R	ACAACGCCTTCCAGCATCG
mvaE-6-F	GCGATGCTGGAAGGCGTTG
mvaE-6-R	GTTCGCCGTCCAGGGTCC
mvaE-7-F	TGGACCCTGGACGGCGAAC
mvaE-R	TTACTGTTTACGCAGGTCGTTC
mvaS-F	ATGACCATTGGCATTGATAAAATCTCT
mvaS-2-R	ACGGGTGGCCGGTCGGA
mvaS-3-R	GCAGAGAAATCAGGCCCAG
mvaS-R	TTAGTTACGGTAAGAACGAACGGTGT
MvaK1-F	ATGAATATCAAGAAGCAAGGTCTGG
MvaK1-2-R	TAGTCTCCGCGATGGCGGT
MvaK1-3-F	ACCGCCATCGCGGAGACTAT
MvaK1-R	TTACTTCTTAACCTCCAGGGACT
MvaK2-F	ATGATCGAAGTTACCACCCCG
MvaK2-2-F	GCAAATACGGTCTGGGTTC
MvaK2-2-R	TTTTTCTTCTTTAGACTGGTG
MvaK2-R	TTAGCATTCTTTCTGGCCGTAGG

mvaD-F	ATGCTGTCTGGTAAAGCGCGT
MvaD-2-R	GCTTCGTGAACCTGAGAC
MvaD-2-F	CTCTGCGTCTGGCCTGGCTG
MvaD-R	TTATTTGTCCATACCTTTGGTTTCGA
idi-F	ATGAACCGCAAAGATGAACACCTG
idi-1-R	CGAAGATCAGACCGTCCG
idi-2-F	AAACCCGGACGGTCTGATC
idi-2-R	GGCCCCAGTCGTCCAGG
idi-3-F	TTTCCTGGACGACTGGGGCCA
idi-R	TTAGCGTTTCGCGAAAACGGTAG
IspA-F	ATGGATTTCCCGCAGCAGCTG
IspA-2-R	CTTTGTCGCCCGCAGACAG
IspA-R	TTATTTGTTACGCTGAATGATATAATCC
crtE-F	ATGACCGTTTGTGCGAAAAAACAC
crtE-2-F	GATATGCCGTGCATGGACG
crtE-2-R	TAGACGCCATCTGCATAGACG
crtE-R	TTAAGAAACCGCCGCCAGTTTTTTAT
crtB-F	ATGGAAGTCGGTTCCAAGTCC
crtB-2-F	CCGGCCTACGCATTTGACC
crtB-R	TTAAACCGGACGCTGCCACAG
crtI-F	ATGAACCGTACGACCGTAATTGG
crtI-2-F	GTGATGTAGAGGGCTATCGC
crtI-2-R	GGCGAGCTACAGACGCGTTC
crtI-3-F	TGAACGCGTCTGTAGCTCG
crtI-3-R	AGCTGCCGCAGCCCTCAG
crtI-R	TTAGGCCAGGTCCTCCAGCAT
T7F-H-F	ACCCAAGCTTGATCTCGATCCCGCGAAATTA
mS-X-R	ACTGTTCTCGAGTTAGTTACGGTAAGAACGAACGGTGT
mk1-N-F	CTAGCTAGCATGAATATCAAGAAGCAAGGTCTGG
ii-H-R	ACTGTTAAGCTTTTAGCGTTTCGCGAAAACGGTAG
iA-N-F	CTAGCTAGCATGGATTTCCCGCAGCAGCTG
T7T-H-R	ACCCAAGCTTGCGGCCGCACTCGAGCACCACCACCACCACCAC
RBS1 [b]	ACGACCTGCGTAAACAGTAAGGAGGATATTTAGATGACCATTGGCATTGATAAAA (5’-3’)<br>TGCTGGACGCATTTGTCATTCCTCCTATAAATCTACTGGTAACCGTAACTATTTT (3’-5’)
RBS2 [b]	CCCTGGAGGTTAAGAAGTAAAAAGAGGAGAAATACTAGATGCTGTCTGGTAAAGCG (5’-3’)<br>GGGACCTCCAATTCTTCATTTTTCTCCTCTTTATGATCTACGACAGACCATTTCGC (3’-5’)
RBS3 [b]	AACCAAAGGTATGGACAAATAAGGAGAAGCATGATCGAAGTTACCACCC (5’-3’)<br>TTGGTTTCCATACCTGTTTATTCCTCTTCGTACTAGCTTCAATGGTGGG (3’-5’)
RBS4 [b]	ACGGCCAGAAAGAATGCTAAGGAGAAGCATGAACCGCAAAGATGAACAC (5’-3’)<br>TGCCGGTCTTTCTTACGATTCCTCTTCGTACTTGGCGTTTCTACTTGTG (3’-5’)
RBS5 [b]	TATATCATTCAGCGTAACAAATAAGGAGGATATTTAGATGACCGTTTGTGCGAAAAA (5’-3’)<br>ATATAGTAAGTCGCATTGTTTATTCCTCCTATAAATCTACTGGCAAACACGCTTTTT (3’-5’)

RBS6 [b]	CTGGAGGACCTGGCCTAAGGAGGATATTTAGATGGAAGTCGGTTCCAAGTC (5'-3')<br>GACCTCCTGGACCGGATTCCTCCTATAAATCTACCTTCAGCCAAGGTTCAG (3'-5')
RBS7 [b]	CTGGCGGCGGTTTCTTAAGGAGGATATTTAGATGAACCGTACGACCGTAAT (5'-3')<br>GACCGCCGCCAAAGAATTCCTCCTATAAATCTACTTGGCATGCTGGCATTA (3'-5')

[a] Underline: the restriction enzyme site

[b] These RBS sequences were dsDNA, and prepared by annealing of the two complementary single strand oligos.

Supplementary Table S2 Primers used in lycopene gene synthesis.

Subpool (pool) Name	Oligos primer (5'->3')		Fragments primer_1[a] (5'->3')		Fragments primer_2[b] (5'->3')	
	forward	reverse	forward	reverse	forward	reverse
mvaE-1	MlyI_1F	MlyI-3F	mvaE-F	Fra-R		
mvaE-2	MlyI_1F	MlyI-4F	Fra-F	mvaE-2-R		
mvaE-3	MlyI_1F	MlyI-5F	mvaE-3-F	Fra-R		
mvaE-4	MlyI_1F	MlyI-6F	Fra-F	mvaE-4-R		
mvaE-5	MlyI_1F	MlyI-7F	mvaE-5-F	Fra-R	mvaE-5-F	mvaE-5-R
mvaE-6	MlyI_1F	MlyI-8F	Fra-F	mvaE-6-R	mvaE-6-F	mvaE-6-R
mvaE-7	MlyI_1F	MlyI-9F	Fra-F	mvaE-R	MvaE-7-F	mvaE-R
mvaS-1	XMlyI_1F	MlyI-3F	mvaS-F	Fra-R		
mvaS-2	XMlyI_1F	MlyI-4F	Fra-F	mvaS-2-R		
mvaS-3	XMlyI_1F	MlyI-5F	Fra-F	mvaS-3-R		
mvaS-4	XMlyI_1F	MlyI-6F	Fra-F	mvaS-R		
mvaK1-1	MlyI_2F	MlyI-3F	MvaK1-F	Fra-R		
mvaK1-2	MlyI_2F	MlyI-4F	Fra-F	MvaK1-2-R		
mvaK1-3	MlyI_2F	MlyI-5F	Fra-F	MvaK1-R	MvaK1-3-F	MvaK1-R
mvaK2-1	XMlyI_2F	MlyI-10F	MvaK2-F	Fra-R		
mvaK2-2	XMlyI_2F	MlyI-11F	MvaK2-2-F	Fra-R	MvaK2-2-F	MvaK2-2-R
mvaK2-3	XMlyI_2F	MlyI-12F	Fra-F	MvaK2-R		
mvaD-1	XMlyI-3F	MlyI-10F	mvaD-F	Fra-R		
mvaD-2	XMlyI-3F	MlyI-11F	Fra-F	MvaD-2-R	MvaD-2-F	MvaD-2-R
mvaD-3	XMlyI-3F	MlyI-12F	Fra-F	MvaD-R		
idi-1	XMlyI-4F	MlyI-12F	idi-F	Fra-R	idi-F	idi-1-R
idi-2	XMlyI-4F	MlyI-13F	idi-2-F	Fra-R	idi-2-F	idi-2-R
idi-3	XMlyI-4F	MlyI-14F	Fra-F	idi-R	idi-3-F	idi-R
ispA-1	XMlyI_1F	MlyI-10F	IspA-F	Fra-R		
ispA-2	XMlyI_1F	MlyI-11F	Fra-F	IspA-2-R		
ispA-3	XMlyI_1F	MlyI-12F	Fra-F	IspA-R		
crtE-1	MlyI_1F	MlyI-10F	crtE-F	Fra-R		
crtE-2	MlyI_1F	MlyI-11F	Fra-F	CrtE-2-R	CrtE-2-F	CrtE-2-R
crtE-3	MlyI_1F	MlyI-12F	Fra-F	CrtE-R		
crtB-1	XMlyI_1F	MlyI-7F	crtB-F	Fra-R		
crtB-2	XMlyI_1F	MlyI-9F	crtB-2-F	Fra-R		
crtB-3	XMlyI_1F	MlyI-8F	Fra-F	crtB-R		
crtI-1	MlyI_2F	MlyI-6F	crtI-F	Fra-R		
crtI-2	MlyI_2F	MlyI-7F	Fra-F	crtI-2-R	crtI-2-F	crtI-2-R
crtI-3	MlyI_2F	MlyI-8F	Fra-F	crtI-3-R	crtI-3-F	crtI-3-R
crtI-4	MlyI_2F	MlyI-10F	Fra-F	crtI-R		

The sequences of primers were listed in Supplementary Table S1.

[a] half-specific primer pair with one specific fragment primer and one F-primer.

[b] full-specific primer pair with two specific fragment primers.

Supplementary table S3 Summary of oligonucleotides design of lycopene biosynthesis genes.

Gene (length：bp)	Fragments (length：bp)	Oligos subpool (length: nt; oligos)
crtB (891)	crtB-F1 (328)	crtB 1 (71-101; 13)
	crtB-F2 (349)	crtB_2 (71-100; 13)
	crtB-F3 (348)	crtB_3 (70-93; 15)
crtE (909)	crtE-F1 (334)	crtE_1 (72-102; 13)
	crtE-F2 (355)	crtE_2 (69-101; 14)
	crtE-F3 (355)	crtE_3 (73-103; 13)
crtI (1479)	crtI-F1 (400)	crtI_1 (72-102; 14)
	crtI-F2 (423)	crtI_2 (72-106; 15)
	crtI-F3 (420)	crtI_3 (65-102; 15)
	crtI-F4 (419)	crtI_4 (70-100; 15)
idi (1044)	idi-F1 (279)	idi_1 (76-113; 13)
	idi-F2 (401)	idi_2 (71-103; 14)
	idi-F3 (397)	idi_3 (72-107; 13)
ispA (900)	ispA-F1 (331)	ispA_1 (65-96; 13)
	ispA-F2 (348)	ispA_2 (66-99; 13)
	ispA-F3 (349)	ispA_3 (69-113; 12)
mvaD (996)	mvaD-F1 (363)	mvaD_1 (66-99; 13)
	mvaD-F2 (381)	mvaD_2 (68-115; 13)
	mvaD-F3 (389)	mvaD_3 (75-115; 13)
mvaE (2412)	mvaE-F1 (375)	mvaE_1 (76-103; 13)
	mvaE-F2 (399)	mvaE_2 (80-119; 13)
	mvaE-F3 (398)	mvaE_3 (72-104; 14)
	mvaE-F4 (397)	mvaE_4 (72-108; 13)
	mvaE-F5 (399)	mvaE_5 (71-104; 14)
	mvaE-F6 (394)	mvaE_6 (71-113; 13)
	mvaE-F7 (396)	mvaE_7 (67-98; 16)
mvaK1 (945)	mvaK1-F1 (346)	mvaK1_1 (71-109; 12)
	mvaK1-F2 (368)	mvaK1_2 (68-105; 13)
	mvaK1-F3 (363)	mvaK1_3 (71-101; 14)
mvaK2 (1083)	mvaK2-F1 (392)	mvaK2_1 (73-116; 13)
	mvaK2-F2 (416)	mvaK2_2 (73-106; 16)
	mvaK2-F3 (419)	mvaK2_3 (77-111; 14)
mvaS (1152)	mvaS-F1 (319)	mvaS_1 (71-113; 10)
	mvaS-F2 (341)	mvaS 2 (64-99; 13)
	mvaS-F3 (334)	mvaS 3 (68-109; 11)
	mvaS-F4 (340)	mvaS 4 (77-124; 11)

Supplementary Table S4 Primers used in the strain WW1 construction.

Primers ID	Sequence[a]
dxr-N-F	CTAGCTAGCATGAAGCAACTCACCATTCTGG
dxr-X-R	CCGCTCGAGTCAGCTTGCGAGACGCATCAC
Tc-A-F	ACTTGGCGCGCCCGATATAAGTTGTAATTCTC
Tc-S-R	TTGGACTAGTTCAGGTCGAGGTGGCC
Pet21c-A-F	CACTTGGCGCGCCCACATTTCCCCGAAAAGTGCCAC
Pet21c-S-R	TTGGACTAGTCTGTCAGACCAAGTTTACTC

[a] Underline: the restriction enzyme site

Supplementary Figures:

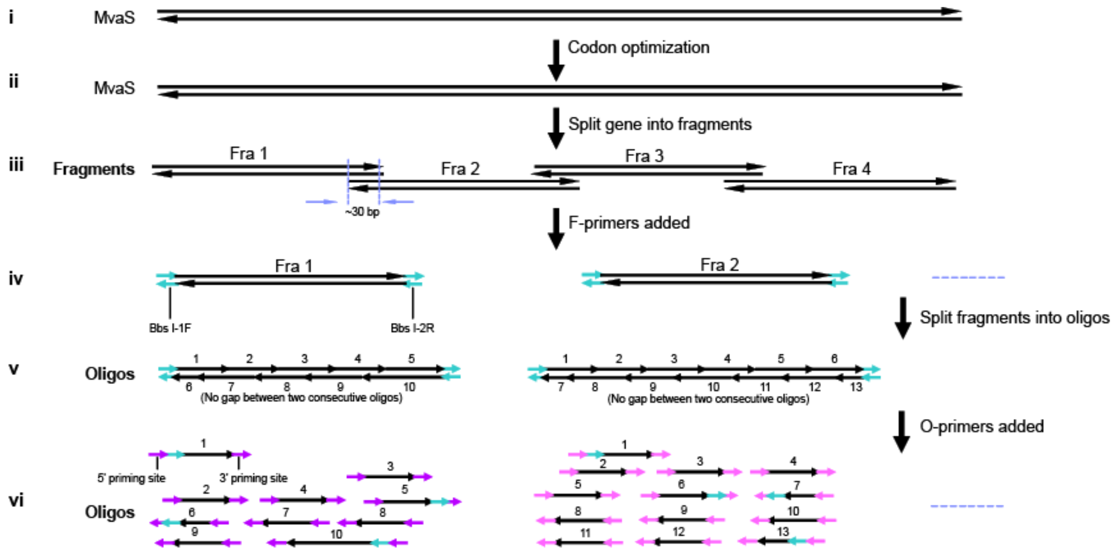

Supplementary Figure S1. Design of oligonucleotides of lycopene biosynthetic pathway genes for synthesis on microchips (*mvaS* oligonucleotides are described as an example).

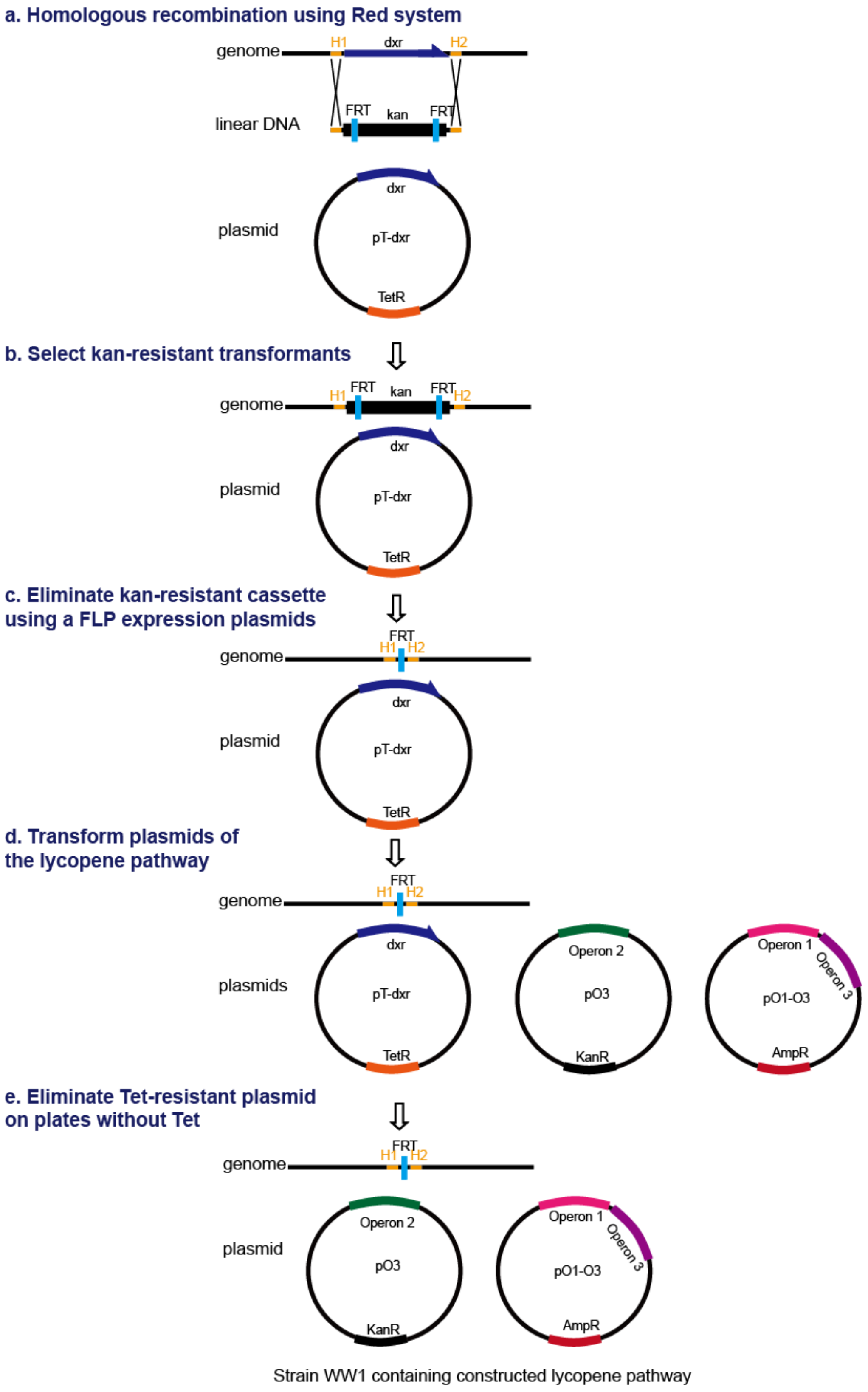

Supplementary Figure S2. Schematic representation of the construction of the WW1 strain harboring the genes of the lycopene biosynthetic pathway.

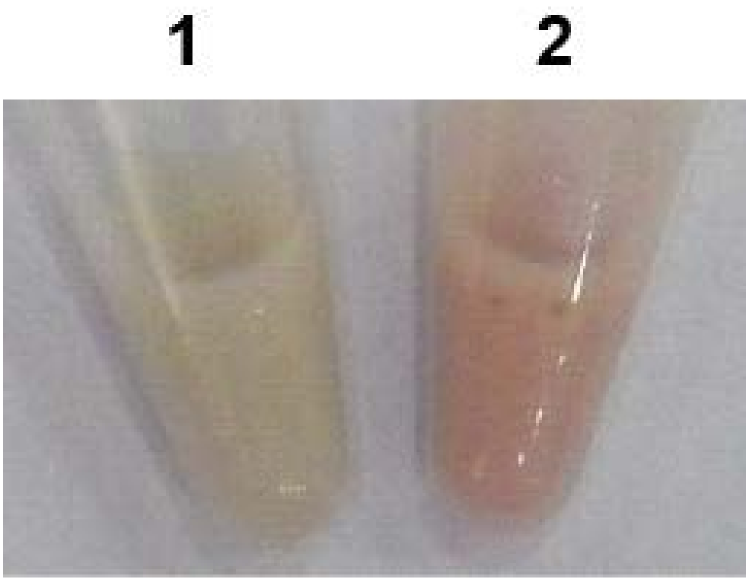

Supplementary Figure S3. Production of lycopene via the synthesized lycopene biosynthetic pathway. 1: JM109 (DE3) harboring pET-21c and pET-28a; 2: strain WW1 harboring pET-O1-O3 and pET-O2.

Supplementary Methods

Construction of *dxr*-deleted *Escherichia coli* WW1 and expression of the synthetic lycopene pathway

The strategy to construct the WW1 strain is illustrated in Supplementary Figure S6. Because D*xr*-disrupted *E. coli* cannot grow without 2-C-methyl-D-erythritol (1), a copy of *dxr* in the pT-dxr plasmid was used to complement the deletion during the deficient strain construction. In brief, the Tc^R gene sequence was PCR-amplified from pACYC184 with Tc-A-F and Tc-S-R primers (Supplementary Table S4) and the pET21c DNA fragment without the *bla* coding sequence was obtained by PCR using the pET21C-A-F and pET21C-S-R primers (Supplementary Table S4). These sequences were double-digested with *Asp*I and *Spe*I and ligated with T4 DNA ligase to generate the plasmid pT-21c. The ORF of *dxr* was amplified from the genome of *E. coli* with the dxr-N-F and dxr-X-R primers (Supplementary Table S4) and cloned into pT-21c *Nhe*I-*Xho*I sites to generate the plasmid pT-dxr. pT-dxr was transformed into *E. coli* JM109 (DE3). Then the chromosomal *dxr* gene sequence was deleted based on the Red system (Supplementary Figure S6) as previously described (2). The *dxr* gene in pT-dxr was under the control of the T7 promoter and leaking expression of this gene was sufficient to support growth. After eliminating the chromosomal *dxr* gene sequence, plasmids pO1-O3 and pO2 were transformed into the constructed strain and transformants were screened on LB medium containing 100 μg/mL ampicillin and 50 μg/mL kanamycin (without tetracycline). Because operon 1 and operon 2 could complement the deficiency of the MEP pathway (blocked in the *dxr* mutant) with the MVA pathway, the pT-dxr-deleted strain could be screened on the medium without tetracycline and confirmed with the loss of Tet^R. The resultant strain harboring the lycopene pathway genes is *dxr* deficient and named WW1.

PCR amplification of oligos

PCR of 50 µL contained 1 ng of template oligos, 100 pmol of primers, 5 µL of 2 mM dNTPs, 3 µL of 25 mM MgSO4, and 1 µL of KOD plus DNA polymerase in 1× reaction buffer. PCR parameters were set as follows: 94°C for 5 min, 30 cycles of 15 s at 94°C, 30 s at 50°C, 30 s at 68°C, and a final extension step of 10 min at 68°C. Amplified oligos were purified with the UNIQ-10 oligonucleotide kit (Sangon Biotech Co. (Shanghai, China)) according to the manufacturer's instructions.

PCR amplification of error-depleted fragments

The 50 µL-PCR mixture contained 5 µL of 10× reaction buffer, 1 µL of 10 mM dNTPs, 2 µL of Pfu DNA polymerase (Biocolor BioScience & Technology Company, Shanghai, China), 1 µL of of eluted fraction, and 2 µL of primer mixture (10 µM). PCR parameters were: 94°C for 30 sec, 50°C for 30 sec, and 72°C for 30 sec for 30 cycles, followed by 72°C for 5 min. The PCR mixture was isolated on 3% (w/v) agarose gels and extracted with the Axygen gel extraction kit (TaKaRa Biotechnology (Dalian) Co. Ltd, Dalian, China) according to the manufacturer's instructions. Products were digested with the type IIS restriction enzyme *Bbs* I to remove the F-primer region for the following full-length DNA assembly. Cleaved short fragment of priming sites were removed with the UNIQ-10 oligonucleotide kit.

Gibson assemble of operons

Aliquots of 20 μL of PCR reaction mixture contained 0.75 μL 10 × Taq DNA ligase buffer, 0.2 μL dNTPs (2.5 mM each), 0.75 μL PEG6000 (50%), 4 μL $MgCl_2$ (25 mM), 0.04 U T5 exonuclease (New England Biolabs, Beverly, MA, USA), 0.25 U phusion High-Fidelity DNA polymerase (New England Biolabs, Beverly, MA, USA), 40 U Taq DNA ligase, and 75 ng DNA. incubate at 50°C for 1 hour.

PCR amplification of full-length operons

The 50 μL-PCR mixture contained 10 μL of 5 × reaction buffer, 4 μL of 2.5 mM dNTPs, 0.5 μL of PrimeSTAR HS DNA polymerase, 1 μL of Gibson assembly product, and 1 μL of each primer (10 μM). PCR parameters were: 98°C for 10 sec, 55°C for 5 sec, and 72°C for 4.5 min for 30 cycles, followed by 72°C for 5 min. The PCR mixture was isolated on 1% (w/v) agarose gels and extracted with the Axygen gel extraction kit according to the manufacturer's instructions.

Supplementary data:

mvaE-1

mvaE-1_1-for

CACAGGAGTCCTCACACGCTCTGAAGACCCATGAAAACCGTTGTTATCATCGACGCGCTGCGCACCC
CGATCGGCAAATACAAAGGCTCTCAGGACTCCTCTG

mvaE-1_2-for

CACAGGAGTCCTCACCTCTGTCTCAGGTTTCTGCGGTTGACCTGGGTACCCACGTTACCACCCAGCTG
CTGCTCAGGACTCCTCTG

mvaE-1_3-for

CACAGGAGTCCTCACAAACGCCACTCTACCATCTCTGAAGAAATCGACCAGGTTATTTTCGGCAACG
TTCTGCAGGCGGCTCAGGACTCCTCTG

mvaE-1_4-for

CACAGGAGTCCTCACGCAACGGTCAGAACCCGGCGCGTCAGATCGCGATCAACTCTGGTCTGTCTCA
CTCAGGACTCCTCTG

mvaE-1_5-for

CACAGGAGTCCTCACCGAAATCCCGGCGATGACCGTTAACGAAGTTTGCGGCTCTGGCATGAAAGC
GGTTATCCTCAGGACTCCTCTG

mvaE-1_6-for

CACAGGAGTCCTCACCTGGCGAAACAGCTGATCCAGCTGGGTGAAGCGGAAGTTCTGATTGCGGGT
GGTATCCTGTCTTCTATCTCGCTCAGGACTCCTCTG

mvaE-1_1-rev

CACAGGAGTCCTCACGCAGCGCGTCGATGATAACAACGGTTTTCATGGGTCTTCAGAGCGTCTCAG
GACTCCTCTG

mvaE-1_2-rev

CACAGGAGTCCTCACCCAGGTCAACCGCAGAAACCTGAGACAGAGAGCCTTTGTATTTGCCGATCG
GGGTGCCTCAGGACTCCTCTG

mvaE-1_3-rev

CACAGGAGTCCTCACTGGTCGATTTCTTCAGAGATGGTAGAGTGGCGTTTCAGCAGCTGGGTGGTA
ACGTGGGTACCTCAGGACTCCTCTG

mvaE-1_4-rev

CACAGGAGTCCTCACGCGCCGGGTTCTGACCGTTGCCCGCCTGCAGAACGTTGCCGAAAATAACCCT
CAGGACTCCTCTG

mvaE-1_5-rev

CACAGGAGTCCTCACTTCGTTAACGGTCATCGCCGGGATTTCGTGAGACAGACCAGAGTTGATCGC
GATCTGACCTCAGGACTCCTCTG

mvaE-1_6-rev

CACAGGAGTCCTCACCCCAGCTGGATCAGCTGTTTCGCCAGGATAACCGCTTTCATGCCAGAGCCGC
AAACCTCAGGACTCCTCTG

mvaE-1_7-rev

CACAGGAGTCCTCACCGAGATAGAAGACAGGATACCACCCGCAATCAGAACTTCCGCTTCACTCAG
GACTCCTCTG

mvaE-2

mvaE-2_1-for
CACAGGAGTCCTCACACGCTCTGAAGACCCGGAAGTTCTGATTGCGGGTGGTATCGAAAACATGTC
TCAGGCGCCGAAACTGCAGCGTTTCGTTGCGACTCGTAGG
mvaE-2_2-for
CACAGGAGTCCTCACAACTACGAAACCGAATCTTACGACGCGCCGTTCTCTTCTATGATGTACGACG
GCCTGACCGACGTTGCGACTCGTAGG
mvaE-2_3-for
CACAGGAGTCCTCACGCGTTCTCTGGTCAGGCGATGGGTCTGACCGCGGAAAACGTTGCGGAAAAA
TATCACGGTTGCGACTCGTAGG
mvaE-2_4-for
CACAGGAGTCCTCACTTACCCGTGAAGAACAGGACCAGTTCTCTGTTCACTCTCAGCTGAAAGCGGC
GCAGGCGTTGCGACTCGTAGG
mvaE-2_5-for
CACAGGAGTCCTCACGCAGGCGGAAGGTATCTTCGCGGATGAAATTGCGCCGCTGGAAGTTTCTGG
CACCGTTGCGACTCGTAGG
mvaE-2_6-for
CACAGGAGTCCTCACCTGGTTGAAAAAGATGAAGGTATTCGCCCGAACTCTTCTGTTGAAAAACTGG
GCACCCTGAAAACCGTTTTCAACTGTCTTCTATCTCGGTTGCGACTCGTAGG
mvaE-2_1-rev
CACAGGAGTCCTCACACATGTTTTCGATACCACCCGCAATCAGAACTTCCGGGTCTTCAGAGCGTGT
TGCGACTCGTAGG
mvaE-2_2-rev
CACAGGAGTCCTCACCGGCGCGTCGTAAGATTCGGTTTCGTAGTTGAAACGCTGCAGTTTCGGCGCC
TGAGGTTGCGACTCGTAGG
mvaE-2_3-rev
CACAGGAGTCCTCACAGACCCATCGCCTGACCAGAGAACGCGTCGGTCAGGCCGTCGTACATCATA
GAAGAGAAGTTGCGACTCGTAGG
mvaE-2_4-rev
CACAGGAGTCCTCACTGAACAGAGAACTGGTCCTGTTCTTCACGGGTAACGTGATATTTTTCCGCAA
CGTTTTCCGCGGTCGTTGCGACTCGTAGG
mvaE-2_5-rev
CACAGGAGTCCTCACAATTTCATCCGCGAAGATACCTTCCGCCTGCGCCTGCGCCGCTTTCAGCTGA
GAGGTTGCGACTCGTAGG
mvaE-2_6-rev
CACAGGAGTCCTCACGAAGAGTTCGGGCGAATACCTTCATCTTTTTCAACCAGGGTGCCAGAAACTT
CCAGCGGCGCGTTGCGACTCGTAGG
mvaE-2_7-rev
CACAGGAGTCCTCACCGAGATAGAAGACAGTTGAAAACGGTTTTCAGGGTGCCCAGTTTTTCAACA
GTTGCGACTCGTAGG

mvaE-3

mvaE-3_1-for
CACAGGAGTCCTCACACGCTCTGAAGACCCGGGCACCCTGAAAACCGTTTTCAAAGAAGATGGCAC
CGTTACCGCGGGTAACGCCAGACGACTCGAATG
mvaE-3_2-for
CACAGGAGTCCTCACGTCTACCATCAACGATGGTGCGTCTGCGCTGATTATTGCGTCTCAGGAATAC
GCGGAAGCCAGACGACTCGAATG
mvaE-3_3-for
CACAGGAGTCCTCACGCACGGTCTGCCGTACCTGGCGATCATTCGCGATTCTGTTGAAGTTGGTATC
GACCCAGACGACTCGAATG
mvaE-3_4-for
CACAGGAGTCCTCACCGGCGTACATGGGTATCTCTCCGATCAAAGCGATCCAGAAACTGCTGGCGC
GCAGACGACTCGAATG
mvaE-3_5-for
CACAGGAGTCCTCACTAACCAGCTGACCACCGAAGAAATCGACCTGTACGAAATCAACGAAGCGTT
CGCGGCCAGACGACTCGAATG
mvaE-3_6-for
CACAGGAGTCCTCACGACCTCTATCGTTGTTCAGCGTGAACTGGCGCTGCCGGAAGAAAAAGTTAA
CATCTACGGTGCAGACGACTCGAATG
mvaE-3_7-for
CACAGGAGTCCTCACGTGGTATCTCTCTGGGTCACGCGATCGCTGTCTTCTATCTCGCAGACGACTC
GAATG
mvaE-3_1-rev
CACAGGAGTCCTCACCATCTTCTTTGAAAACGGTTTTCAGGGTGCCCGGGTCTTCAGAGCGTCAGAC
GACTCGAATG
mvaE-3_2-rev
CACAGGAGTCCTCACCGCAGACGCACCATCGTTGATGGTAGACGCGTTACCCGCGGTAACGGTGCC
AGACGACTCGAATG
mvaE-3_3-rev
CACAGGAGTCCTCACGCCAGGTACGGCAGACCGTGCGCTTCCGCGTATTCCTGAGACGCAATAATC
AGCAGACGACTCGAATG
mvaE-3_4-rev
CACAGGAGTCCTCACTTTGATCGGAGAGATACCCATGTACGCCGGGTCGATACCAACTTCAACAGAA
TCGCGAATGATCCAGACGACTCGAATG
mvaE-3_5-rev
CACAGGAGTCCTCACAGGTCGATTTCTTCGGTGGTCAGCTGGTTACGCGCCAGCAGTTTCTGGATCG
CCAGACGACTCGAATG
mvaE-3_6-rev
CACAGGAGTCCTCACGCCAGTTCACGCTGAACAACGATAGAGGTCGCCGCGAACGCTTCGTTGATTT
CGTACCAGACGACTCGAATG
mvaE-3_7-rev
CACAGGAGTCCTCACCGAGATAGAAGACAGCGATCGCGTGACCCAGAGAGATACCACCACCGTAGA
TGTTAACTTTTTCTTCCGGCAGCCAGACGACTCGAATG

mvaE-4

mvaE-4_1-for

CACAGGAGTCCTCACACGCTCTGAAGACCCTATCTCTCTGGGTCACGCGATCGGTGCGACCGGTGCG
CGTCTGCTGACCGAAGGGACTCGTACG

mvaE-4_2-for

CACAGGAGTCCTCACTCTCTGTCTTACCAGCTGAACCAGAAAGAAAAAAAATACGGCGTTGCGTCTC
TGTGCATTGGTGGCGGGAAGGGACTCGTACG

mvaE-4_3-for

CACAGGAGTCCTCACTCTGGGTCTGGCGATGCTGCTGGAACGCCCGCAGCAGAAAAAAAACTCTCG
CTTCTACGAAGGGACTCGTACG

mvaE-4_4-for

CACAGGAGTCCTCACCAGATGTCTCCGGAAGAACGTCTGGCGTCTCTGCTGAACGAAGGTCAGATC
TCTGCGGAGAAGGGACTCGTACG

mvaE-4_5-for

CACAGGAGTCCTCACCACCAAAAAAGAATTCGAAAACACCGCGCTGTCCTCTCAGATCGCGAACCAC
ATGATCGAAAACCAGATTGAAGGGACTCGTACG

mvaE-4_6-for

CACAGGAGTCCTCACTCTGAAACCGAAGTTCCGATGGGTGTTGGTCTGCACCTGACCGTTGACGAAA
CCGACTATCTGCTGTCTTCTATCTCGGAAGGGACTCGTACG

mvaE-4_1-rev

CACAGGAGTCCTCACGCACCGATCGCGTGACCCAGAGAGATAGGGTCTTCAGAGCGTGAAGGGAC
TCGTACG

mvaE-4_2-rev

CACAGGAGTCCTCACGCCGTATTTTTTTTCTTTCTGGTTCAGCTGGTAAGACAGAGAGGTCAGCAGA
CGCGCACCGGTCGAAGGGACTCGTACG

mvaE-4_3-rev

CACAGGAGTCCTCACGTTCCAGCAGCATCGCCAGACCCAGACCGCCACCAATGCACAGAGACGCAA
CGAAGGGACTCGTACG

mvaE-4_4-rev

CACAGGAGTCCTCACACGCCAGACGTTCTTCCGGAGACATCTGGTAGAAGCGAGAGTTTTTTTTCTG
CTGCGGGCGAAGGGACTCGTACG

mvaE-4_5-rev

CACAGGAGTCCTCACGGACAGCGCGGTGTTTTCGAATTCTTTTTTGGTGTCCGCAGAGATCTGACCT
TCGTTCAGCAGAGGAAGGGACTCGTACG

mvaE-4_6-rev

CACAGGAGTCCTCACAGACCAACACCCATCGGAACTTCGGTTTCAGAAATCTGGTTTTCGATCATGT
GGTTCGCGATCTGAGAGAAGGGACTCGTACG

mvaE-4_7-rev

CACAGGAGTCCTCACCGAGATAGAAGACAGCAGATAGTCGGTTTCGTCAACGGTCAGGTGCGAAG
GGACTCGTACG

mvaE-5

mvaE-5_1-for

CACAGGAGTCCTCACACGCTCTGAAGACCCGACCGTTGACGAAACCGACTATCTGGTTCCGATGGC GACCGAAGAACCGTCTGTTATCGATACAGACTCGACAG

mvaE-5_2-for

CACAGGAGTCCTCACCGGCGCTGTCTAACGGTGCGAAAATCGCGCAGGGTTTCAAAACCGTTAACC AGATACAGACTCGACAG

mvaE-5_3-for

CACAGGAGTCCTCACCAGCGTCTGATGCGTGGCCAGATTGTTTTCTACGACGTTGCGGACCCGGAAT CATACAGACTCGACAG

mvaE-5_4-for

CACAGGAGTCCTCACTCTGATCGACAAACTGCAGGTTCGTGAAGCGGAAATTTTCCAGCAGGCGGA ACTGTCTTACATACAGACTCGACAG

mvaE-5_5-for

CACAGGAGTCCTCACCCGTCTATCGTTAAACGTGGTGGTGGTCTGCGCGACCTGCAGTACCGTGCGA TACAGACTCGACAG

mvaE-5_6-for

CACAGGAGTCCTCACTTCGATGAATCTTTCATCTCTGTTGACTTCCTGGTTGACGTTAAAGATGCGAT GGGCGCGAACATCATACAGACTCGACAG

mvaE-5_7-for

CACAGGAGTCCTCACGTTAACGCGATGCTGGAAGGCGTTGTCTGTCTTCTATCTCGATACAGACTCG ACAG

mvaE-5_1-rev

CACAGGAGTCCTCACGGAACCAGATAGTCGGTTTCGTCAACGGTCGGGTCTTCAGAGCGTATACAG ACTCGACAG

mvaE-5_2-rev

CACAGGAGTCCTCACATTTTCGCACCGTTAGACAGCGCCGCGATAACAGACGGTTCTTCGGTCGCCA TCATACAGACTCGACAG

mvaE-5_3-rev

CACAGGAGTCCTCACCAATCTGGCCACGCATCAGACGCTGCTGGTTAACGGTTTTGAAACCCTGCGC GATACAGACTCGACAG

mvaE-5_4-rev

CACAGGAGTCCTCACGCTTCACGAACCTGCAGTTTGTCGATCAGAGATTCCGGGTCCGCAACGTCGT AGAAAAATACAGACTCGACAG

mvaE-5_5-rev

CACAGGAGTCCTCACCAGACCACCACCACGTTTAACGATAGACGGGTAAGACAGTTCCGCCTGCTG GAAAATTTCCATACAGACTCGACAG

mvaE-5_6-rev

CACAGGAGTCCTCACTCAACCAGGAAGTCAACAGAGATGAAAGATTCATCGAACGCACGGTACTGC AGGTCGCGATACAGACTCGACAG

mvaE-5_7-rev

CACAGGAGTCCTCACCGAGATAGAAGACAGACAACGCCTTCCAGCATCGCGTTAACGATGTTCGCG CCCATCGCATCTTTAACGATACAGACTCGACAG

mvaE-6

mvaE-6_1-for

CACAGGAGTCCTCACACGCTCTGAAGACCCGCGATGCTGGAAGGCGTTGTTGAACTGTTCCGTGAATGGTTCGCGGAACAGAAAATCCTGTTCTCTATGATCGGACTCGTACC

mvaE-6_2-for

CACAGGAGTCCTCACCCTGTCTAACTACGCGACCGAATCTGTTGTTACCATGAAAACCGCGATTCCGGTTTCTCGCCTGTGATCGGACTCGTACC

mvaE-6_3-for

CACAGGAGTCCTCACCTAAAGGTTCTAACGGCCGTGAAATCGCGGAAAAAATTGTTCTGGCGTCTCGTTACGCGTCTCTGGAGATCGGACTCGTACC

mvaE-6_4-for

CACAGGAGTCCTCACCCCGTACCGTGCGGTTACCCACAACAAAGGCATCATGAACGGCATTGAAGCGGTTGTTCGATCGGACTCGTACC

mvaE-6_5-for

CACAGGAGTCCTCACTGGCGACCGGTAACGACACCCGTGCGGTTTCTGCGTCTTGCCACGCGTTCGATCGGACTCGTACC

mvaE-6_6-for

CACAGGAGTCCTCACGCGGTTAAAGAAGGCCGTTACCAGGGTCTGACCTCTTGGACCCTGGACGGCGAACCTGTCTTCTATCTCGGATCGGACTCGTACC

mvaE-6_1-rev

CACAGGAGTCCTCACGAACAGTTCAACAACGCCTTCCAGCATCGCGGGTCTTCAGAGCGTGATCGGACTCGTACC

mvaE-6_2-rev

CACAGGAGTCCTCACCATGGTAACAACAGATTCGGTCGCGTAGTTAGACAGGATAGAGAACAGGATTTTCTGTTCCGCGAACCATTCACGGATCGGACTCGTACC

mvaE-6_3-rev

CACAGGAGTCCTCACCAATTTTTTCCGCGATTTCACGGCCGTTAGAACCTTTAGACAGGCGAGAAACCGGAATCGCGGTTTTGATCGGACTCGTACC

mvaE-6_4-rev

CACAGGAGTCCTCACTTTGTTGTGGGTAACCGCACGGTACGGGTCCAGAGACGCGTAACGAGACGCCAGAAGATCGGACTCGTACC

mvaE-6_5-rev

CACAGGAGTCCTCACCACGGGTGTCGTTACCGGTCGCCAGAACAACCGCTTCAATGCCGTTCATGATGCCGATCGGACTCGTACC

mvaE-6_6-rev

CACAGGAGTCCTCACAGACCCTGGTAACGGCCTTCTTTAACCGCGAACGCGTGGCAAGACGCAGAAACCGGATCGGACTCGTACC

mvaE-6_7-rev

CACAGGAGTCCTCACCGAGATAGAAGACAGGTTCGCCGTCCAGGGTCCAAGAGGTCGATCGGACTCGTACC

mvaE-7

mvaE-7_1-for
CACAGGAGTCCTCACACGCTCTGAAGACCCTGGACCCTGGACGGCGAACAGCTGATTGGTGAAATT TCTGTTCCGCTGGCGGAGACGACTCGTAAG
mvaE-7_2-for
CACAGGAGTCCTCACCTGGCGACCGTTGGCGGTGCGACCAAAGTTCTGCCGAAATCTCAGGCGGAG ACGACTCGTAAG
mvaE-7_3-for
CACAGGAGTCCTCACGCGGCGGACCTGCTGGCGGTTACCGACGCGAAAGAACTGTCTCGTGTGAGA CGACTCGTAAG
mvaE-7_4-for
CACAGGAGTCCTCACTGTTGCGGCGGTTGGTCTGGCGCAGAACCTGGCGGCGCTGCGAGACGACTC GTAAG
mvaE-7_5-for
CACAGGAGTCCTCACGCGCGCTGGTTTCTGAAGGTATCCAGAAAGGTCACATGGCGCTGCAGGCGA GACGACTCGTAAG
mvaE-7_6-for
CACAGGAGTCCTCACGCGTTCTCTGGCGATGACCGTTGGCGCGACCGGCAAAGAAGTTGAAGCGGA GACGACTCGTAAG
mvaE-7_7-for
CACAGGAGTCCTCACGTTGCGCAGCAGCTGAAACGTCAGAAAACCATGAACCAGGACCGTGCGCTG GAGACGACTCGTAAG
mvaE-7_8-for
CACAGGAGTCCTCACGCGATCCTGAACGACCTGCGTAAACAGTAACTGTCTTCTATCTCGGAGACGA CTCGTAAG
mvaE-7_1-rev
CACAGGAGTCCTCACGCTGTTCGCCGTCCAGGGTCCAGGGTCTTCAGAGCGTGAGACGACTCGTAA G
mvaE-7_2-rev
CACAGGAGTCCTCACGCACCGCCAACGGTCGCCAGCGCCAGCGGAACAGAAATTTCACCAATCAGA GACGACTCGTAAG
mvaE-7_3-rev
CACAGGAGTCCTCACCGCCAGCAGGTCCGCCGCCGCCTGAGATTTCGGCAGAACTTTGGTCGAGAC GACTCGTAAG
mvaE-7_4-rev
CACAGGAGTCCTCACGCCAGACCAACCGCCGCAACAACACGAGACAGTTCTTTCGCGTCGGTAACG AGACGACTCGTAAG
mvaE-7_5-rev
CACAGGAGTCCTCACTCTGGATACCTTCAGAAACCAGCGCGCGCAGCGCCGCCAGGTTCTGCGAGA CGACTCGTAAG
mvaE-7_6-rev
CACAGGAGTCCTCACCCAACGGTCATCGCCAGAGAACGCGCCTGCAGCGCCATGTGACCTTGAGAC GACTCGTAAG

mvaE-7_7-rev
CACAGGAGTCCTCACTTTTCTGACGTTTCAGCTGCTGCGCAACCGCTTCAACTTCTTTGCCGGTCGCG
GAGACGACTCGTAAG
mvaE-7_8-rev
CACAGGAGTCCTCACCGAGATAGAAGACAGTTACTGTTTACGCAGGTCGTTCAGGATCGCCAGCGC
ACGGTCCTGGTTCATGGGAGACGACTCGTAAG

mvaS-1
mvaS-1_1-for
GTATAGAGTCAGTCGACGCTCTGAAGACCCATGACCATTGGCATTGATAAAATCTCTTTCTTCGTTCC
GCCGTACTACATCGACATGACCGCGCTGGCCTCAGGACTCCTCTG
mvaS-1_2-for
GTATAGAGTCAGTCGGGAAGCGCGTAACGTTGATCCGGGTAAATTCCACATCGGTATCGGTCAGGA
CCAGATGGCCTCAGGACTCCTCTG
mvaS-1_3-for
GTATAGAGTCAGTCGGGTTAACCCGATTTCTCAGGATATTGTTACCTTCGCGGCGAACGCGGCGGA
AGCGATCCCTCAGGACTCCTCTG
mvaS-1_4-for
GTATAGAGTCAGTCGTGACCAAAGAAGATAAAGAAGCGATTGACATGGTTATCGTTGGCACCGAAT
CTTCTATCGACGAATCTAAAGCGGCCTCAGGACTCCTCTG
mvaS-1_5-for
GTATAGAGTCAGTCGGGCGGTTGTTCTGCACCGTCTGATGGCTGTCTTCTATCTCGCTCAGGACTCCT
CTG
mvaS-1_1-rev
GTATAGAGTCAGTCGGCGGAACGAAGAAAGAGATTTTATCAATGCCAATGGTCATGGGTCTTCAGA
GCGTCTCAGGACTCCTCTG
mvaS-1_2-rev
GTATAGAGTCAGTCGGAATTTACCCGGATCAACGTTACGCGCTTCCGCCAGCGCGGTCATGTCGATG
TAGTACGCTCAGGACTCCTCTG
mvaS-1_3-rev
GTATAGAGTCAGTCGCGCGAAGGTAACAATATCCTGAGAAATCGGGTTAACCGCCATCTGGTCCTG
ACCGATACCGATGTGCTCAGGACTCCTCTG
mvaS-1_4-rev
GTATAGAGTCAGTCGCAACGATAACCATGTCAATCGCTTCTTTATCTTCTTTGGTCAGGATCGCTTCC
GCCGCGTTCGCCTCAGGACTCCTCTG
mvaS-1_5-rev
GTATAGAGTCAGTCGCGAGATAGAAGACAGCCATCAGACGGTGCAGAACAACCGCCGCCGCTTTAG
ATTCGTCGATAGAAGATTCGGTGCCTCAGGACTCCTCTG

mvaS-2
mvaS-2_1-for
GTATAGAGTCAGTCGACGCTCTGAAGACCCGGTTGTTCTGCACCGTCTGATGGGTATTCAGCCGTTC
GCGCGTTCTTTCGAAATGTTGCGACTCGTAGG

mvaS-2_2-for
GTATAGAGTCAGTCGCAAAGAAGCGTGCTACGGTGCGACCGCGGGTCTGCAGCTGGCGAAGTTGC
GACTCGTAGG
mvaS-2_3-for
GTATAGAGTCAGTCGAAACCACGTTGCGCTGCACCCGGACAAAAAAGTTCTGGTTGTTGCGGCGGA
TATCGTTGCGACTCGTAGG
mvaS-2_4-for
GTATAGAGTCAGTCGGCGAAATATGGTCTGAACTCTGGTGGTGAACCGACCCAGGGTGCGGGTGC
GTTGCGACTCGTAGG
mvaS-2_5-for
GTATAGAGTCAGTCGGGTTGCGATGCTGGTTGCGTCTGAACCGCGTATTCTGGCGCTGAAAGAAGA
TAACGTTATGTTGCGACTCGTAGG
mvaS-2_6-for
GTATAGAGTCAGTCGGCTGACCCAGGACATCTACGACTTCTGGCGTCCGACCGGCCACCCGTCTGTC
TTCTATCTCGGTTGCGACTCGTAGG
mvaS_2_1-rev
GTATAGAGTCAGTCGGAATACCCATCAGACGGTGCAGAACAACCGGGTCTTCAGAGCGTGTTGCGA
CTCGTAGG
mvaS_2_2-rev
GTATAGAGTCAGTCGGGTCGCACCGTAGCACGCTTCTTTGATTTCGAAAGAACGCGCGAACGGCTG
TTGCGACTCGTAGG
mvaS_2_3-rev
GTATAGAGTCAGTCGCGGGTGCAGCGCAACGTGGTTTTTCGCCAGCTGCAGACCCGCGTTGCGACT
CGTAGG
mvaS_2_4-rev
GTATAGAGTCAGTCGGTTCACCACCAGAGTTCAGACCATATTTCGCGATATCCGCCGCAACAACCAG
AACTTTTTTGTCGTTGCGACTCGTAGG
mvaS_2_5-rev
GTATAGAGTCAGTCGGTTCAGACGCAACCAGCATCGCAACCGCACCCGCACCCTGGGTCGGTTGCG
ACTCGTAGG
mvaS_2_6-rev
GTATAGAGTCAGTCGCCAGAAGTCGTAGATGTCCTGGGTCAGCATAACGTTATCTTCTTTCAGCGCC
AGAATACGCGGTTGCGACTCGTAGG
mvaS_2_7-rev
GTATAGAGTCAGTCGCGAGATAGAAGACAGACGGGTGGCCGGTCGGACGGTTGCGACTCGTAGG

mvaS-3
mvaS-3_1-for
GTATAGAGTCAGTCGACGCTCTGAAGACCCCCGACCGGCCACCCGTATCCGATGGTTGACGGTCCG
CTGTCTAACGAAACCTACATCAGACGACTCGAATG
mvaS-3_2-for
GTATAGAGTCAGTCGCCAGTCTTTCGCGCAGGTTTGGGACGAACACAAAAAACGCACCGGTCTGGA
CTTCGCCAGACGACTCGAATG

mvaS-3_3-for
GTATAGAGTCAGTCGGGACTACGATGCGCTGGCGTTCCACATTCCGTACACCAAAATGGGCAAAAA AGCGCTGCTCAGACGACTCGAATG
mvaS-3_4-for
GTATAGAGTCAGTCGGGCGAAAATTTCTGACCAGACCGAAGCGGAACAGGAACGTATCCTGGCGC GTTACGAAGAATCTATCCAGACGACTCGAATG
mvaS-3_5-for
GTATAGAGTCAGTCGGTTTATTCTCGTCGCGTTGGCAACCTGTACACCGGTTCTCTGTACCTGGGCCT GATTTCTCTGCCTGTCTTCTATCTCGCAGACGACTCGAATG
mvaS-3_1-rev
GTATAGAGTCAGTCGATCGGATACGGGTGGCCGGTCGGGGGTCTTCAGAGCGTCAGACGACTCGA ATG
mvaS-3_2-rev
GTATAGAGTCAGTCGTTCGTCCCAAACCTGCGCGAAAGACTGGATGTAGGTTTCGTTAGACAGCGG ACCGTCAACCCAGACGACTCGAATG
mvaS-3_3-rev
GTATAGAGTCAGTCGGAATGTGGAACGCCAGCGCATCGTAGTCCGCGAAGTCCAGACCGGTGCGTT TTTTGTGCAGACGACTCGAATG
mvaS-3_4-rev
GTATAGAGTCAGTCGTTCCGCTTCGGTCTGGTCAGAAATTTTCGCCAGCAGCGCTTTTTTGCCCATTT TGGTGTACGCAGACGACTCGAATG
mvaS-3_5-rev
GTATAGAGTCAGTCGGGTGTACAGGTTGCCAACGCGACGAGAATAAACGATAGATTCTTCGTAACG CGCCAGGATACGTTCCTGCAGACGACTCGAATG
mvaS-3_6-rev
GTATAGAGTCAGTCGCGAGATAGAAGACAGGCAGAGAAATCAGGCCCAGGTACAGAGAACCCAGA CGACTCGAATG

mvaS-4
mvaS-4_1-for
GTATAGAGTCAGTCGACGCTCTGAAGACCCGTACCTGGGCCTGATTTCTCTGCTGGAAAACGCGACC ACCCTGACCGCGGGTAACCGAAGGGACTCGTACG
mvaS-4_2-for
GTATAGAGTCAGTCGAGATCGGTCTGTTCTCTTACGGCTCTGGTGCGGTTGCGGAATTCTTCACCGG TGAACTGGTGAAGGGACTCGTACG
mvaS-4_3-for
GTATAGAGTCAGTCGTGCGGGTTATCAGAACCACCTGCAGAAAGAAACCCACCTGGCGCTGCTGGA CAACCGAAGGGACTCGTACG
mvaS-4_4-for
GTATAGAGTCAGTCGGTACCGAACTGTCTATCGCGGAATACGAAGCGATGTTCGCGGAAACCCTGG ACACCGAGAAGGGACTCGTACG

mvaS-4_5-for

GTATAGAGTCAGTCGCATCGACCAGACCCTGGAAGATGAACTGAAATACTCTATCTCTGCGATCAAC AACACCGTTCGTTCTTACCGTAACTAACTGTCTTCTATCTCGGAAGGGACTCGTACG

mvaS-4_1-rev

GTATAGAGTCAGTCGCGTTTTCCAGCAGAGAAATCAGGCCCAGGTACGGGTCTTCAGAGCGTGAAG GGACTCGTACG

mvaS-4_2-rev

GTATAGAGTCAGTCGGCACCAGAGCCGTAAGAGAACAGACCGATCTGGTTACCCGCGGTCAGGGT GGTCGGAAGGGACTCGTACG

mvaS-4_3-rev

GTATAGAGTCAGTCGGTTTCTTTCTGCAGGTGGTTCTGATAACCCGCAACCAGTTCACCGGTGAAGA ATTCCGCAACCGAAGGGACTCGTACG

mvaS-4_4-rev

GTATAGAGTCAGTCGTCGCTTCGTATTCCGCGATAGACAGTTCGGTACGGTTGTCCAGCAGCGCCAG GTGGGAAGGGACTCGTACG

mvaS-4_5-rev

GTATAGAGTCAGTCGAGATAGAGTATTTCAGTTCATCTTCCAGGGTCTGGTCGATGTCGGTGTCCAG GGTTTCCGCGAACAGAAGGGACTCGTACG

mvaS-4_6-rev

GTATAGAGTCAGTCGCGAGATAGAAGACAGTTAGTTACGGTAAGAACGAACGGTGTTGTTGATCGC AGGAAGGGACTCGTACG

mvaK1-1

mvaK1-1_1-for

CCAAGGAGTCGCTAGACGCTCTGAAGACCCATGAATATCAAGAAGCAAGGTCTGGGCCAGGCGACC GGTAAAATCATTCTGATGGGTGAGCACGCTCAGGACTCCTCTG

mvaK1-1_2-for

CCAAGGAGTCGCTAGCGGTCGTTTACGGTGAACCTGCGATTGCGTTCCCGTTCCAAGCGACTGAAAT TACCGCCTCAGGACTCCTCTG

mvaK1-1_3-for

CCAAGGAGTCGCTAGTGTGTTCACGCCGGCAAAGACCATGCAGATCGACTGCGCGTACTTCACGGG TCTCAGGACTCCTCTG

mvaK1-1_4-for

CCAAGGAGTCGCTAGCTGCTGGAAGATGTCCCGCAGGAACTGGCGAACATCAAGGAGGTGGTGCA ACAAACCCTCAGGACTCCTCTG

mvaK1-1_5-for

CCAAGGAGTCGCTAGCTGCACTTCCTGAAGGAAGATACGTTCAAGGGCACCCTGACGCTGACGTCT ACCATTCCCTCAGGACTCCTCTG

mvaK1-1_6-for

CCAAGGAGTCGCTAGGGCGGAGCGTGGTATGGGTTCTTCTGCTGTCTTCTATCTCGCTCAGGACTCC TCTG

mvaK1-1_1-rev
CCAAGGAGTCGCTAGGCCTGGCCCAGACCTTGCTTCTTGATATTCATGGGTCTTCAGAGCGTCTCAG
GACTCCTCTG
mvaK1-1_2-rev
CCAAGGAGTCGCTAGGCAATCGCAGGTTCACCGTAAACGACCGCGTGCTCACCCATCAGAATGATTT
TACCGGTCCTCAGGACTCCTCTG
mvaK1-1_3-rev
CCAAGGAGTCGCTAGGCATGGTCTTTGCCGGCGTGAACACAGCGGTAATTTCAGTCGCTTGGAACG
GGAACCTCAGGACTCCTCTG
mvaK1-1_4-rev
CCAAGGAGTCGCTAGCAGTTCCTGCGGGACATCTTCCAGCAGACCCGTGAAGTACGCGCAGTCGAT
CTCTCAGGACTCCTCTG
mvaK1-1_5-rev
CCAAGGAGTCGCTAGCCCTTGAACGTATCTTCCTTCAGGAAGTGCAGGGTTTGTTGCACCACCTCCTT
GATGTTCGCCTCAGGACTCCTCTG
mvaK1-1_6-rev
CCAAGGAGTCGCTAGCGAGATAGAAGACAGCAGAAGAACCCATACCACGCTCCGCCGGAATGGTA
GACGTCAGCGTCAGGGTGCTCAGGACTCCTCTG

mvaK1-2
mvaK1-2_1-for
CCAAGGAGTCGCTAGACGCTCTGAAGACCCGGAGCGTGGTATGGGTTCTTCTGCGGCGACGGCGGT
GGCGATCGTACGCGTTGCGACTCGTAGG
mvaK1-2_2-for
CCAAGGAGTCGCTAGTCCCTGTTTGACTACTTCGATTACGCCTACACGTACCAAGAGCTGTTCGAGC
TGGTTTCTCTGTCTGAAAAGATCGTTGCGACTCGTAGG
mvaK1-2_3-for
CCAAGGAGTCGCTAGGCACACGGTAACCCGTCTGGCATCGACGCGGCTGCCACGTCCGGTGGTTGC
GACTCGTAGG
mvaK1-2_4-for
CCAAGGAGTCGCTAGCGGACCCGCTGTTCTTCACCCGTGGTTTCCCGCCGACCCACTTCTCTATGAAT
CTGTGTTGCGACTCGTAGG
mvaK1-2_5-for
CCAAGGAGTCGCTAGCTAACGCGTACCTGGTCGTTGCGGACACCGGTATCAAAGGTCAAACCCGCG
AGGCGGTTGCGACTCGTAGG
mvaK1-2_6-for
CCAAGGAGTCGCTAGGTTAAGGACATCGCCCAACTGGCTCAGAACAACCCGACCGCCATCGCGGAG
ACTACTGTCTTCTATCTCGGTTGCGACTCGTAGG
mvaK1-2_1-rev
CCAAGGAGTCGCTAGGCCGCAGAAGAACCCATACCACGCTCCGGGTCTTCAGAGCGTGTTGCGACT
CGTAGG

mvaK1-2_2-rev
CCAAGGAGTCGCTAGGTACGTGTAGGCGTAATCGAAGTAGTCAAACAGGGAGCGTACGATCGCCA
CCGCCGTCGTTGCGACTCGTAGG
mvaK1-2_3-rev
CCAAGGAGTCGCTAGTCGATGCCAGACGGGTTACCGTGTGCGATCTTTTCAGACAGAGAAACCAGC
TCGAACAGCTCTTGGTTGCGACTCGTAGG
mvaK1-2_4-rev
CCAAGGAGTCGCTAGCACGGGTGAAGAACAGCGGGTCCGCACCGGACGTGGCAGCCGCGGTTGCG
ACTCGTAGG
mvaK1-2_5-rev
CCAAGGAGTCGCTAGGTGTCCGCAACGACCAGGTACGCGTTAGACAGATTCATAGAGAAGTGGGTC
GGCGGGAAACGTTGCGACTCGTAGG
mvaK1-2_6-rev
CCAAGGAGTCGCTAGTTGTTCTGAGCCAGTTGGGCGATGTCCTTAACCGCCTCGCGGGTTTGACCTT
TGATACCGGTTGCGACTCGTAGG
mvaK1-2_7-rev
CCAAGGAGTCGCTAGCGAGATAGAAGACAGTAGTCTCCGCGATGGCGGTCGGGGTTGCGACTCGT
AGG

mvaK1-3
mvaK1-3_1-for
CCAAGGAGTCGCTAGACGCTCTGAAGACCCACCGCCATCGCGGAGACTATGAAACAGCTGGGCTCT
TTCACCAAGGAAGCACAGACGACTCGAATG
mvaK1-3_2-for
CCAAGGAGTCGCTAGAAGCAAGCGATCCTGCAAGACGACAAGCAGAAACTGGGTCAACTGATGAC
CCTGGCAGACGACTCGAATG
mvaK1-3_3-for
CCAAGGAGTCGCTAGCGCAGGAGCAACTGCAGCAGCTGACCGTTTCTAACGACATGCTGGACCGCA
GACGACTCGAATG
mvaK1-3_4-for
CCAAGGAGTCGCTAGCCTGGTAGCGCTGTCTCTGGAACACGGTGCGCTGGGTGCGAAACTGACAGA
CGACTCGAATG
mvaK1-3_5-for
CCAAGGAGTCGCTAGCCGGTGGTGGTCGTGGCGGTTGCATGATTGCCCTGACCGATAACAAGAAAA
CAGACGACTCGAATG
mvaK1-3_6-for
CCAAGGAGTCGCTAGCTGCACAGACCATTGCCCAAACGCTGGAGGAGAACGGTGCCGTTGCCAGAC
GACTCGAATG
mvaK1-3_7-for
CCAAGGAGTCGCTAGGACTTGGATTCAGTCCCTGGAGGTTAAGAAGTAACTGTCTTCTATCTCGCAG
ACGACTCGAATG

mvaK1-3_1-rev
CCAAGGAGTCGCTAGTGTTTCATAGTCTCCGCGATGGCGGTGGGTCTTCAGAGCGTCAGACGACTC
GAATG
mvaK1-3_2-rev
CCAAGGAGTCGCTAGCTTGTCGTCTTGCAGGATCGCTTGCTTTGCTTCCTTGGTGAAAGAGCCCAGC
CAGACGACTCGAATG
mvaK1-3_3-rev
CCAAGGAGTCGCTAGAGCTGCTGCAGTTGCTCCTGCGCCAGGGTCATCAGTTGACCCAGTTTCTGCA
GACGACTCGAATG
mvaK1-3_4-rev
CCAAGGAGTCGCTAGGTGTTCCAGAGACAGCGCTACCAGGCGGTCCAGCATGTCGTTAGAAACGGT
CCAGACGACTCGAATG
mvaK1-3_5-rev
CCAAGGAGTCGCTAGCCGCCACGACCACCACCGGTCAGTTTCGCACCCAGCGCACCCAGACGACTC
GAATG
mvaK1-3_6-rev
CCAAGGAGTCGCTAGGCGTTTGGGCAATGGTCTGTGCAGTTTTCTTGTTATCGGTCAGGGCAATCAT
GCAACAGACGACTCGAATG
mvaK1-3_7-rev
CCAAGGAGTCGCTAGCGAGATAGAAGACAGTTACTTCTTAACCTCCAGGGACTGAATCCAAGTCGC
AACGGCACCGTTCTCCTCCACAGACGACTCGAATG

mvaK2-1
mvaK2-1_1-for
GTCACGAGTCATGGCACGCTCTGAAGACCCATGATCGAAGTTACCACCCCGGGTAAACTGTTCATTG
CGGGCGAATATGCGGTTGTTGAAGTAGAGACTCGATCC
mvaK2-1_2-for
GTCACGAGTCATGGCCCGGGTCACCCGGCGATCATTGTTGCGGTTGATCAGTTCGTTACCGTTACCG
TTGGTAGAGACTCGATCC
mvaK2-1_3-for
GTCACGAGTCATGGCAAGAAACCACCGATGAAGGTTCTATCCAGTCTGCGCAGTACTCTTCTCTGCC
GATCCGCTGGTAGAGACTCGATCC
mvaK2-1_4-for
GTCACGAGTCATGGCGACCCGTCGTAACGGCGAACTGGTTCTGGACATCCGCGAAAACCCGTTCCA
CTAGTAGAGACTCGATCC
mvaK2-1_5-for
GTCACGAGTCATGGCCGTTCTGGCGGCGATTCACCTGACCGAAAAATACGCGCAGGAACAGAACAA
AGAACTGTCTGTAGAGACTCGATCC
mvaK2-1_6-for
GTCACGAGTCATGGCTTCTATCACCTGAAAGTTACCTCTGAACTGGATTCTTCTAACGGCCGCAAATA
CGGTCTGGGTTCTTCTGGCTGTCTTCTATCTCGGTAGAGACTCGATCC

mvaK2-1_1-rev

GTCACGAGTCATGGCACAGTTTACCCGGGGTGGTAACTTCGATCATGGGTCTTCAGAGCGTGTAGA GACTCGATCC

mvaK2-1_2-rev

GTCACGAGTCATGGCAACAATGATCGCCGGGTGACCCGGTTCAACAACCGCATATTCGCCCGCAAT GAGTAGAGACTCGATCC

mvaK2-1_3-rev

GTCACGAGTCATGGCGCAGACTGGATAGAACCTTCATCGGTGGTTTCTTCAACGGTAACGGTAACG AACTGATCAACCGCGTAGAGACTCGATCC

mvaK2-1_4-rev

GTCACGAGTCATGGCGAACCAGTTCGCCGTTACGACGGGTCCAGCGGATCGGCAGAGAAGAGTAC TGCGTAGAGACTCGATCC

mvaK2-1_5-rev

GTCACGAGTCATGGCGGTCAGGTGAATCGCCGCCAGAACGTAGTGGAACGGGTTTTCGCGGATGTC CAGTAGAGACTCGATCC

mvaK2-1_6-rev

GTCACGAGTCATGGCCGTTAGAAGAATCCAGTTCAGAGGTAACTTTCAGGTGATAGAAAGACAGTT CTTTGTTCTGTTCCTGCGCGTATTTTTCGTAGAGACTCGATCC

mvaK2-1_7-rev

GTCACGAGTCATGGCCGAGATAGAAGACAGCCAGAAGAACCCAGACCGTATTTGCGGCGTAGAGA CTCGATCC

mvaK2-2

mvaK2-2_1-for

GTCACGAGTCATGGCACGCTCTGAAGACCCGCAAATACGGTCTGGGTTCTTCTGGCGCGGTTACCGT TGGCACCGTTAAAGCGCTCATATGACTCCACAC

mvaK2-2_2-for

GTCACGAGTCATGGCGAACATTTTCTACGACCTGGGCCTGGAAAACGAAGAAATTTTCAAACTGTCT GCGCTGGCGCACATATGACTCCACAC

mvaK2-2_3-for

GTCACGAGTCATGGCCCTGGCGGTTCAGGGCAACGGTTCTTGCGGTGACATCGCGGCGTCATATGA CTCCACAC

mvaK2-2_4-for

GTCACGAGTCATGGCCTTGCTACGGTGGCTGGATCGCGTTCTCTACCTTCGACCACGACTGGGTTAA CCCATATGACTCCACAC

mvaK2-2_5-for

GTCACGAGTCATGGCAGAAAGTTGCGACCGAAACCCTGACCGACCTGCTGGCGATGGACTGGCCCA TATGACTCCACAC

mvaK2-2_6-for

GTCACGAGTCATGGCGGAACTGATGATCTTCCCGCTGAAAGTTCCGAAACAGCTGCGTCTGCTGATC GGTTGGACCATATGACTCCACAC

mvaK2-2_7-for

GTCACGAGTCATGGCCGGCTCTCCGGCGTCTACCTCTGACCTGGTTGACCGTGTTCACCAGTCTAAAGAAGAAAAACTGTCTTCTATCTCGCATATGACTCCACAC

mvaK2-2_1-rev

GTCACGAGTCATGGCGCGCCAGAAGAACCCAGACCGTATTTGCGGGTCTTCAGAGCGTCATATGACTCCACAC

mvaK2-2_2-rev

GTCACGAGTCATGGCCGTTTTCCAGGCCCAGGTCGTAGAAAATGTTCAGCGCTTTAACGGTGCCAACGGTAACCCATATGACTCCACAC

mvaK2-2_3-rev

GTCACGAGTCATGGCACCGTTGCCCTGAACCGCCAGGTGCGCCAGCGCAGACAGTTTGAAAATTTCTTCATATGACTCCACAC

mvaK2-2_4-rev

GTCACGAGTCATGGCACGCGATCCAGCCACCGTAGCAAGACGCCGCGATGTCACCGCAAGACATATGACTCCACAC

mvaK2-2_5-rev

GTCACGAGTCATGGCCGGTCAGGGTTTCGGTCGCAACTTTCTGGTTAACCCAGTCGTGGTCGAAGGTAGAGACATATGACTCCACAC

mvaK2-2_6-rev

GTCACGAGTCATGGCTTTCGGAACTTTCAGCGGGAAGATCATCAGTTCCGGCCAGTCCATCGCCAGCAGGTCATATGACTCCACAC

mvaK2-2_7-rev

GTCACGAGTCATGGCTCAGAGGTAGACGCCGGAGAGCCGGTCCAACCGATCAGCAGACGCAGCTGCATATGACTCCACAC

mvaK2-2_8-rev

GTCACGAGTCATGGCCGAGATAGAAGACAGTTTTTCTTCTTTAGACTGGTGAACACGGTCAACCAGGCATATGACTCCACAC

mvaK2-3

mvaK2-3_1-for

GTCACGAGTCATGGCACGCTCTGAAGACCCACCGTGTTCACCAGTCTAAAGAAGAAAAACAGGCGGCGTACGAACAGTTCCTGATGAAATCTCGTCGAATTGACTCGATCC

mvaK2-3_2-for

GTCACGAGTCATGGCTGTGCGTTGAAACCATGATCAACGGCTTCAACACCGGCAAAATCTCTGTTATCCAGAAACAGATTACCGAATTGACTCGATCC

mvaK2-3_3-for

GTCACGAGTCATGGCAAAAACCGTCAGCTGCTGGCGGAACTGTCCTCTCTGACCGGCGTTGTTATCGAAACCGAATTGACTCGATCC

mvaK2-3_4-for

GTCACGAGTCATGGCGAAGCGCTGAAAAACCTGTGCGACCTGGCGGAATCTTATACCGGTGCGGCGAAATCGAATTGACTCGATCC

mvaK2-3_5-for

GTCACGAGTCATGGCTTCTGGTGCGGGTGGCGGTGATTGCGGTATCGTTATCTTCCGTCAGAAATCT GGTATCCTGGAATTGACTCGATCC

mvaK2-3_6-for

GTCACGAGTCATGGCCCGCTGATGACCGCGTGGGAAAAAGACGGCATTACCCCGCTGCCGCTGAAT TGACTCGATCC

mvaK2-3_7-for

GTCACGAGTCATGGCGCACGTTTACACCTACGGCCAGAAAGAATGCTAACTGTCTTCTATCTCGGAA TTGACTCGATCC

mvaK2-3_1-rev

GTCACGAGTCATGGCGCCTGTTTTTCTTCTTTAGACTGGTGAACACGGTGGGTCTTCAGAGCGTGAA TTGACTCGATCC

mvaK2-3_2-rev

GTCACGAGTCATGGCTGAAGCCGTTGATCATGGTTTCAACGCACAGACGAGATTTCATCAGGAACT GTTCGTACGCCGAATTGACTCGATCC

mvaK2-3_3-rev

GTCACGAGTCATGGCCAGTTCCGCCAGCAGCTGACGGTTTTTGGTAATCTGTTTCTGGATAACAGAG ATTTTGCCGGTGTGAATTGACTCGATCC

mvaK2-3_4-rev

GTCACGAGTCATGGCCAGGTCGCACAGGTTTTTCAGCGCTTCGGTTTCGATAACAACGCCGGTCAGA GAGGAGAATTGACTCGATCC

mvaK2-3_5-rev

GTCACGAGTCATGGCCAATCACCGCCACCCGCACCAGAAGATTTCGCCGCACCGGTATAAGATTCCG CGAATTGACTCGATCC

mvaK2-3_6-rev

GTCACGAGTCATGGCTCTTTTTCCCACGCGGTCATCAGCGGCAGGATACCAGATTTCTGACGGAAGA TAACGATACCGGAATTGACTCGATCC

mvaK2-3_7-rev

GTCACGAGTCATGGCCGAGATAGAAGACAGTTAGCATTCTTTCTGGCCGTAGGTGTAAACGTGCAG CGGCAGCGGGGTAATGCCGGAATTGACTCGATCC

mvaD-1

mvaD-1_1-for

GATAAGAGTCTCAGCACGCTCTGAAGACCCATGCTGTCTGGTAAAGCGCGTGCGCACACCAACATC GCGCTGATCAAATACTGGGTAGAGACTCGATCC

mvaD-1_2-for

GATAAGAGTCTCAGCGGCAAAGCGAACGAAGAATACATCCTGCCGATGAACTCTTCTCTGTCTCTGA CCCTGGATGGTAGAGACTCGATCC

mvaD-1_3-for

GATAAGAGTCTCAGCCGTTCTACACCGAAACCACCGTTACCTTCGACGCGCACTACTCTGAAGATGT TTTCATCCGTAGAGACTCGATCC

mvaD-1_4-for
GATAAGAGTCTCAGCTGGACGGCATCCTGCAGAACGAGAAACAGACCAAGAAAGTTAAAGAATTCC
TGAACCTGGTGTAGAGACTCGATCC
mvaD-1_5-for
GATAAGAGTCTCAGCTCGCCAGCAGGCGGATTGTACCTGGTTCGCGAAAGTTGAATCTCAGAACTTC
GTTCGTAGAGACTCGATCC
mvaD-1_6-for
GATAAGAGTCTCAGCCGACCGCAGCTGGTCTGGCGTCCTCTGCGTCTGGCCTGGCTCTGTCTTCTAT
CTCGGTAGAGACTCGATCC
mvaD-1_1-rev
GATAAGAGTCTCAGCCGCACGCGCTTTACCAGACAGCATGGGTCTTCAGAGCGTGTAGAGACTCGA
TCC
mvaD-1_2-rev
GATAAGAGTCTCAGCGGCAGGATGTATTCTTCGTTCGCTTTGCCCCAGTATTTGATCAGCGCGATGT
TGGTGTGGTAGAGACTCGATCC
mvaD-1_3-rev
GATAAGAGTCTCAGCGAAGGTAACGGTGGTTTCGGTGTAGAACGCATCCAGGGTCAGAGACAGAG
AAGAGTTCATCGTAGAGACTCGATCC
mvaD-1_4-rev
GATAAGAGTCTCAGCTCTCGTTCTGCAGGATGCCGTCCAGGATGAAAACATCTTCAGAGTAGTGCGC
GTCGTAGAGACTCGATCC
mvaD-1_5-rev
GATAAGAGTCTCAGCAGGTACAATCCGCCTGCTGGCGAACCAGGTTCAGGAATTCTTTAACTTTCTT
GGTCTGTTGTAGAGACTCGATCC
mvaD-1_6-rev
GATAAGAGTCTCAGCCGCCAGACCAGCTGCGGTCGGAACGAAGTTCTGAGATTCAACTTTCGCGAA
CCGTAGAGACTCGATCC
mvaD-1_7-rev
GATAAGAGTCTCAGCCGAGATAGAAGACAGAGCCAGGCCAGACGCAGAGGAGTAGAGACTCGATC
C

mvaD-2
mvaD-2_1-for
GATAAGAGTCTCAGCACGCTCTGAAGACCCCTCTGCGTCTGGCCTGGCTGCACTGGCGGGTGCGTG
CAACGTTGCCATATGACTCCACAC
mvaD-2_2-for
GATAAGAGTCTCAGCGCTGGGTCTGAACCTGTCTGCGAAAGACCTGTCTCGTCTGGCGCGTCGTGG
TCATATGACTCCACAC
mvaD-2_3-for
GATAAGAGTCTCAGCTCTGGTTCTGCGTGCCGTTCTATCTTCGGTGGTTTCGCGCAGTGGAACAAAG
GTCACTCATATGACTCCACAC

mvaD-2_4-for
GATAAGAGTCTCAGCCTGACGAAACCTCTTTCGCGGAAAACATCCCGGCGAACAACTGGGAAAACG AACTGGCGATGCATATGACTCCACAC
mvaD-2_5-for
GATAAGAGTCTCAGCCTGTTCATCCTGATCAACGACGGTGAAAAAGACGTTTCTTCTCGCGACGGCA TGAAACGTACCGCATATGACTCCACAC
mvaD-2_6-for
GATAAGAGTCTCAGCTTGAAACCTCTTCTTTCTACCAGGGCTGGCTGGACAACGTTGAAAAAGATCT GTCTCAGGTTCACGAAGCCTGTCTTCTATCTCGCATATGACTCCACAC
mvaD-2_1-rev
GATAAGAGTCTCAGCGTGCAGCCAGGCCAGACGCAGAGGGGTCTTCAGAGCGTCATATGACTCCAC AC
mvaD-2_2-rev
GATAAGAGTCTCAGCGGTCTTTCGCAGACAGGTTCAGACCCAGCGCAACGTTGCACGCACCCGCCA CATATGACTCCACAC
mvaD-2_3-rev
GATAAGAGTCTCAGCCCGAAGATAGAACGGCACGCAGAACCAGAACCACGACGCGCCAGACGAGA CACATATGACTCCACAC
mvaD-2_4-rev
GATAAGAGTCTCAGCCGGGATGTTTTCCGCGAAAGAGGTTTCGTCAGAGTGACCTTTGTTCCACTGC GCGAAACCACATATGACTCCACAC
mvaD-2_5-rev
GATAAGAGTCTCAGCACGTCTTTTTCACCGTCGTTGATCAGGATGAACAGCATCGCCAGTTCGTTTTC CCAGTTGTTCGCCATATGACTCCACAC
mvaD-2_6-rev
GATAAGAGTCTCAGCCAGCCAGCCCTGGTAGAAAGAAGAGGTTTCAACGGTACGTTTCATGCCGTC GCGAGAAGAACATATGACTCCACAC
mvaD-2_7-rev
GATAAGAGTCTCAGCCGAGATAGAAGACAGGCTTCGTGAACCTGAGACAGATCTTTTTCAACGTTGT CCATATGACTCCACAC

mvaD-3
mvaD-3_1-for
GATAAGAGTCTCAGCACGCTCTGAAGACCCAAAAAGATCTGTCTCAGGTTCACGAAGCGATTAAAA CCAAAGACTTCCCGCGCCTGGGTGAAATCATTGAGAATTGACTCGATCC
mvaD-3_2-for
GATAAGAGTCTCAGCAGCGAACGGCCTGCGTATGCACGGTACCACCCTGGGCGCGGTTCCGAATTG ACTCGATCC
mvaD-3_3-for
GATAAGAGTCTCAGCGCCGTTCACCTACTGGTCTCCGGGTTCTCTGCAGGCGATGGCGCTGGTTGAA TTGACTCGATCC

mvaD-3_4-for

GATAAGAGTCTCAGCCGCCAGGCGCGTGCGAAAGGTATCCCGTGTTACTTCACCATGGACGCTGGT CGAATTGACTCGATCC

mvaD-3_5-for

GATAAGAGTCTCAGCCGAACGTTAAAGTTCTGGTTGAAAAGAAGAACCTGGAAGCGCTGAAAACCT TCCTGTCTGAACACTTCTCTAAAGAACAGGAATTGACTCGATCC

mvaD-3_6-for

GATAAGAGTCTCAGCCTGGTTCCGGCGTTCGCGGGTCCGGGTATCGAACTGTTCGAAACCAAAGGT ATGGACAAATAACTGTCTTCTATCTCGGAATTGACTCGATCC

mvaD-3_1-rev

GATAAGAGTCTCAGCCTTTGGTTTTAATCGCTTCGTGAACCTGAGACAGATCTTTTTGGGTCTTCAGA GCGTGAATTGACTCGATCC

mvaD-3_2-rev

GATAAGAGTCTCAGCCGTGCATACGCAGGCCGTTCGCTTCAATGATTTCACCCAGGCGCGGGAAGT GAATTGACTCGATCC

mvaD-3_3-rev

GATAAGAGTCTCAGCACCCGGAGACCAGTAGGTGAACGGCGGAACCGCGCCCAGGGTGGTACGAA TTGACTCGATCC

mvaD-3_4-rev

GATAAGAGTCTCAGCATACCTTTCGCACGCGCCTGGCGAACCAGCGCCATCGCCTGCAGAGAGAAT TGACTCGATCC

mvaD-3_5-rev

GATAAGAGTCTCAGCGCTTCCAGGTTCTTCTTTTCAACCAGAACTTTAACGTTCGGACCAGCGTCCAT GGTGAAGTAACACGGGGAATTGACTCGATCC

mvaD-3_6-rev

GATAAGAGTCTCAGCGACCCGCGAACGCCGGAACCAGCTGTTCTTTAGAGAAGTGTTCAGACAGGA AGGTTTTCAGCGAATTGACTCGATCC

mvaD-3_7-rev

GATAAGAGTCTCAGCCGAGATAGAAGACAGTTATTTGTCCATACCTTTGGTTTCGAACAGTTCGATA CCCGGAATTGACTCGATCC

idi-1

idi-1_1-for

GAGTGGAGTCTACCGACGCTCTGAAGACCCATGAACCGCAAAGATGAACACCTGTCTCTGGCGAAA GCGTTCCACAAAAAAAAATCTAACGACTTCGAGAATTGACTCGATCC

idi-1_2-for

GAGTGGAGTCTACCGCCGTGTTCGTTTCGTTCACCAGTCTTTCGCGGAATCTGCGGTTAACGAAGTT GACATCTCTGAATTGACTCGATCC

idi-1_3-for

GAGTGGAGTCTACCGACCTCTTTCCTGTCTTTCCAGCTGCCGCAGCCGTTCTATGTTAACGCGATGAC CGGGAATTGACTCGATCC

idi-1_4-for
GAGTGGAGTCTACCGCGGTTCTCAGCGTGCGAAAGAAATCAACCAGCAGCTGGGTATCATTGCGAA
AGAAACCGGAATTGACTCGATCC
idi-1_5-for
GAGTGGAGTCTACCGGCCTGCTGGTTGCGACCGGTTCTGTTTCTGCGGCGCTGAAAGACGCGAATT
GACTCGATCC
idi-1_6-for
GAGTGGAGTCTACCGGTCTCTGGCGGACACCTACCAGATCATGCGTAAAGAAAACCCGGACGGTCT
GATCTTCGCTGTCTTCTATCTCGGAATTGACTCGATCC
idi-1_1-rev
GAGTGGAGTCTACCGCCAGAGACAGGTGTTCATCTTTGCGGTTCATGGGTCTTCAGAGCGTGAATT
GACTCGATCC
idi-1_2-rev
GAGTGGAGTCTACCGCGAAAGACTGGTGAACGAAACGAACACGGTCGAAGTCGTTAGATTTTTTTT
TGTGGAACGCTTTCGGAATTGACTCGATCC
idi-1_3-rev
GAGTGGAGTCTACCGCGGCAGCTGGAAAGACAGGAAAGAGGTAGAGATGTCAACTTCGTTAACCG
CAGATTCCGGAATTGACTCGATCC
idi-1_4-rev
GAGTGGAGTCTACCGGGTTGATTTCTTTCGCACGCTGAGAACCGCCGGTCATCGCGTTAACATAGAA
CGGCTGGAATTGACTCGATCC
idi-1_5-rev
GAGTGGAGTCTACCGGAACCGGTCGCAACCAGCAGGCCGGTTTCTTTCGCAATGATACCCAGCTGC
TGAATTGACTCGATCC
idi-1_6-rev
GAGTGGAGTCTACCGCATGATCTGGTAGGTGTCCGCCAGAGACGCGTCTTTCAGCGCCGCAGAAAC
AGAATTGACTCGATCC
idi-1_7-rev
GAGTGGAGTCTACCGCGAGATAGAAGACAGCGAAGATCAGACCGTCCGGGTTTTCTTTACGGAATT
GACTCGATCC

idi-2
idi-2_1-for
GAGTGGAGTCTACCGACGCTCTGAAGACCCAAACCCGGACGGTCTGATCTTCGCGAACATCGGTGC
GGGTCTGGGTGTTGATGGATGACTCGAACG
idi-2_2-for
GAGTGGAGTCTACCGAGAAGCGAAACGTGCGCTGGACCTGTTCCAGGCGAACGCGCTGCAGATCT
GGATGACTCGAACG
idi-2_3-for
GAGTGGAGTCTACCGCACGTTAACGTTCCGCAGGAACTGGTTATGCCGGAAGGCGATCGTGACTTC
ACCAACTGTGGATGACTCGAACG

idi-2_4-for
GAGTGGAGTCTACCGGCTGACCAAAATCGAAGCGATCGTTCAGGCGGTTGAAGTTCCGGTTATTGT
TAAAGAAGTTGGCTTCGTGGATGACTCGAACG
idi-2_5-for
GAGTGGAGTCTACCGGCATGTCTCAGGAAACCCTGGAAAAACTGACCTCTATCGGCGTTCAGGCGG
CGGATGGATGACTCGAACG
idi-2_6-for
GAGTGGAGTCTACCGCGTTTCTGGTCAGGGTGGTACCTCTTTCACCCAGATCGAAAACGCGCGTCGT
AAAAAACGTGATGGATGACTCGAACG
idi-2_7-for
GAGTGGAGTCTACCGACTGTCTTTCCTGGACGACTGGGGCCCTGTCTTCTATCTCGTGGATGACTCG
AACG
idi-2_1-rev
GAGTGGAGTCTACCGTCGCGAAGATCAGACCGTCCGGGTTTGGGTCTTCAGAGCGTTGGATGACTC
GAACG
idi-2_2-rev
GAGTGGAGTCTACCGCAGGTCCAGCGCACGTTTCGCTTCTTCAACACCCAGACCCGCACCGATGTTG
GATGACTCGAACG
idi-2_3-rev
GAGTGGAGTCTACCGGCATAACCAGTTCCTGCGGAACGTTAACGTGGATCTGCAGCGCGTTCGCCT
GGAATGGATGACTCGAACG
idi-2_4-rev
GAGTGGAGTCTACCGGCCTGAACGATCGCTTCGATTTTGGTCAGCCAGTTGGTGAAGTCACGATCG
CCTTCCGTGGATGACTCGAACG
idi-2_5-rev
GAGTGGAGTCTACCGGGTCAGTTTTTCCAGGGTTTCCTGAGACATGCCGAAGCCAACTTCTTTAACA
ATAACCGGAACTTCAACCTGGATGACTCGAACG
idi-2_6-rev
GAGTGGAGTCTACCGGGTGAAAGAGGTACCACCCTGACCAGAAACGTCCGCCGCCTGAACGCCGAT
AGATGGATGACTCGAACG
idi-2_7-rev
GAGTGGAGTCTACCGCGAGATAGAAGACAGGGCCCCAGTCGTCCAGGAAAGACAGTTCACGTTTTT
TACGACGCGCGTTTTCGATCTGTGGATGACTCGAACG

idi-3
idi-3_1-for
GAGTGGAGTCTACCGACGCTCTGAAGACCCTTTCCTGGACGACTGGGGCCAGTCTACCGTTATCTCT
CTGCTGGAATCTCAGAACTGGCAGCATCCGACTCCATGC
idi-3_2-for
GAGTGGAGTCTACCGAAAAAACTGACCATTCTGGGTTCTGGTGGCGTTCGTAACTCTCTGGACATCG
TTAAAGGCCTGGCCATCCGACTCCATGC

idi-3_3-for
GAGTGGAGTCTACCGGCTGGGCGCGAAATCTATGGGCGTTGCGGGTACCATTCTGGCGTCTCTGATGTCATCCGACTCCATGC
idi-3_4-for
GAGTGGAGTCTACCGCTAAAAACGGTCTGGAAAACACCCTGGCGCTGGTTCAGCAGTGGCAGGAAGAAGTTAAAATGCTCATCCGACTCCATGC
idi-3_5-for
GAGTGGAGTCTACCGGTATACCCTGCTGGGCAAAAAAACCACCGAAGAACTGACCTCTACCGCGCTGGTTCTGGATCCATCCGACTCCATGC
idi-3_6-for
GAGTGGAGTCTACCGCGGTTCTGGTTAACTGGTGCCACAACCGTGGCATCGATTCTACCGTTTTCGCGAAACGCTAACTGTCTTCTATCTCGCATCCGACTCCATGC
idi-3_1-rev
GAGTGGAGTCTACCGGTAGACTGGCCCCAGTCGTCCAGGAAAGGGTCTTCAGAGCGTCATCCGACTCCATGC
idi-3_2-rev
GAGTGGAGTCTACCGAACGCCACCAGAACCCAGAATGGTCAGTTTTTCTGCCAGTTCTGAGATTCCAGCAGAGAGATAACGCATCCGACTCCATGC
idi-3_3-rev
GAGTGGAGTCTACCGAACGCCCATAGATTTCGCGCCCAGCGCCAGGCCTTTAACGATGTCCAGAGAGTTACGCATCCGACTCCATGC
idi-3_4-rev
GAGTGGAGTCTACCGGCGCCAGGGTGTTTTCCAGACCGTTTTTAGACATCAGAGACGCCAGAATGGTACCCGCCATCCGACTCCATGC
idi-3_5-rev
GAGTGGAGTCTACCGTTCTTCGGTGGTTTTTTTGCCCAGCAGGGTATACAGCATTTTAACTTCTTCCTGCCACTGCTGAACCACATCCGACTCCATGC
idi-3_6-rev
GAGTGGAGTCTACCGACGGTTGTGGCACCAGTTAACCAGAACCGGATCCAGAACCAGCGCGGTAGAGGTCAGCATCCGACTCCATGC
idi-3_7-rev
GAGTGGAGTCTACCGCGAGATAGAAGACAGTTAGCGTTTCGCGAAAACGGTAGAATCGATGCCCATCCGACTCCATGC

crtE-1
crtE-1_1-for
CACAGGAGTCCTCACACGCTCTGAAGACCCATGACCGTTTGTGCGAAAAAACACGTTCACCTGACCCGTGATGCGGCGGAACAGCGTAGAGACTCGATCC
crtE-1_2-for
CACAGGAGTCCTCACTGCTGGCGGACATCGACCGTCGTCTGGATCAGCTGCTGCCGGTTGAAGGGTAGAGACTCGATCC

crtE-1_3-for
CACAGGAGTCCTCACTGAACGTGATGTTGTTGGTGCGGCGATGCGCGAAGGTGCGCTGGCGCGTA GAGACTCGATCC
crtE-1_4-for
CACAGGAGTCCTCACCGGGCAAACGTATCCGTCCGATGCTGCTGCTGCTGACCGCGCGCGAGTAGA GACTCGATCC
crtE-1_5-for
CACAGGAGTCCTCACTCTGGGTTGTGCGGTTTCTCACGATGGCCTGCTGGATCTGGCGTGCGCGGG TAGAGACTCGATCC
crtE-1_6-for
CACAGGAGTCCTCACTTGAAATGGTTCACGCGGCGTCTCTGATCCTGGACGATATGCCGTGCATGGA CGACGCTGTCTTCTATCTCGGTAGAGACTCGATCC
crtE-1_1-rev
CACAGGAGTCCTCACAGGTGAACGTGTTTTTTCGCACAAACGGTCATGGGTCTTCAGAGCGTGTAGA GACTCGATCC
crtE-1_2-rev
CACAGGAGTCCTCACACGACGGTCGATGTCCGCCAGCAGCTGTTCCGCCGCATCACGGGTCGTAGA GACTCGATCC
crtE-1_3-rev
CACAGGAGTCCTCACCATCGCCGCACCAACAACATCACGTTCACCTTCAACCGGCAGCAGCTGATCC AGGTAGAGACTCGATCC
crtE-1_4-rev
CACAGGAGTCCTCACCAGCATCGGACGGATACGTTTGCCCGGCGCCAGCGCACCTTCGCGGTAGAG ACTCGATCC
crtE-1_5-rev
CACAGGAGTCCTCACGCCATCGTGAGAAACCGCACAACCCAGATCGCGCGCGGTCAGCAGCAGGTA GAGACTCGATCC
crtE-1_6-rev
CACAGGAGTCCTCACGGATCAGAGACGCCGCGTGAACCATTTCAACCGCGCACGCCAGATCCAGCA GGTAGAGACTCGATCC
crtE-1_7-rev
CACAGGAGTCCTCACCGAGATAGAAGACAGCGTCGTCCATGCACGGCATATCGTCCAGTAGAGACT CGATCC

crtE-2
crtE-2_1-for
CACAGGAGTCCTCACACGCTCTGAAGACCCGATATGCCGTGCATGGACGACGCGAAACTGCGCCGT GGTCGCCCCATATGACTCCACAC
crtE-2_2-for
CACAGGAGTCCTCACGACCATTCACTCTCACTACGGCGAACACGTTGCGATTCTGGCGGCGGTTGCA TATGACTCCACAC

crtE-2_3-for
CACAGGAGTCCTCACCGCTGCTGTCTAAAGCGTTCGGTGTTATCGCGGATGCGGACGGTCTGACCAT
ATGACTCCACAC
crtE-2_4-for
CACAGGAGTCCTCACCCCGCTGGCGAAAAACCGTGCGGTTTCTGAACTGTCTAACGCGATCGGTATG
CATATGACTCCACAC
crtE-2_5-for
CACAGGAGTCCTCACCAGGGCCTGGTTCAGGGCCAGTTCAAAGATCTGTCTGAAGGCGACAAACCG
CATATGACTCCACAC
crtE-2_6-for
CACAGGAGTCCTCACCGCTCTGCGGAAGCGATCCTGATGACCAACCACTTCAAAACCTCTACCCTGT
TCCATATGACTCCACAC
crtE-2_7-for
CACAGGAGTCCTCACTGCGCGTCTATGCAGATGGCGTCTACTGTCTTCTATCTCGCATATGACTCCAC
AC
crtE-2_1-rev
CACAGGAGTCCTCACCGCGTCGTCCATGCACGGCATATCGGGTCTTCAGAGCGTCATATGACTCCAC
AC
crtE-2_2-rev
CACAGGAGTCCTCACGTGTTCGCCGTAGTGAGAGTGAATGGTCGGGCGACCACGGCGCAGTTTCAT
ATGACTCCACAC
crtE-2_3-rev
CACAGGAGTCCTCACATAACACCGAACGCTTTAGACAGCAGCGCAACCGCCGCCAGAATCGCAACC
ATATGACTCCACAC
crtE-2_4-rev
CACAGGAGTCCTCACGCACGGTTTTTCGCCAGCGGGGTCAGACCGTCCGCATCCGCGCATATGACTC
CACAC
crtE-2_5-rev
CACAGGAGTCCTCACTGGCCCTGAACCAGGCCCTGCATACCGATCGCGTTAGACAGTTCAGAAACCC
ATATGACTCCACAC
crtE-2_6-rev
CACAGGAGTCCTCACATCAGGATCGCTTCCGCAGAGCGCGGTTTGTCGCCTTCAGACAGATCTTTGA
ACCATATGACTCCACAC
crtE-2_7-rev
CACAGGAGTCCTCACCGAGATAGAAGACAGTAGACGCCATCTGCATAGACGCGCAGAACAGGGTA
GAGGTTTTGAAGTGGTTGGTCCATATGACTCCACAC

crtE-3
crtE-3_1-for
CACAGGAGTCCTCACACGCTCTGAAGACCCCGCGTCTATGCAGATGGCGTCTATTGTTGCGAACGCG
TCCTCTGAAGCGCGTGATTGGAATTGACTCGATCC

crtE-3_2-for
CACAGGAGTCCTCACCCTGCACCGTTTCTCTCTGGACCTGGGCCAGGCGTTCCAGCTGCTGGACGGA
ATTGACTCGATCC
crtE-3_3-for
CACAGGAGTCCTCACATCTGACCGATGGCATGACCGACACCGGCAAAGATTCTAACCAGGACGCGG
GTAAATCTACCGAATTGACTCGATCC
crtE-3_4-for
CACAGGAGTCCTCACCTGGTTAACCTGCTGGGTCCGCGTGCGGTTGAAGAACGTCTGCGTCAGCAC
CTGAATTGACTCGATCC
crtE-3_5-for
CACAGGAGTCCTCACGCAGCTGGCGTCTGAACACCTGTCTGCGGCGTGCCAGCACGGCCAGAATTG
ACTCGATCC
crtE-3_6-for
CACAGGAGTCCTCACCGCGACCCAGCACTTCATCCAGGCGTGGTTCGATAAAAAACTGGCGGCGGT
TTCTTAACTGTCTTCTATCTCGGAATTGACTCGATCC
crtE-3_1-rev
CACAGGAGTCCTCACGCAACAATAGACGCCATCTGCATAGACGCGGGGTCTTCAGAGCGTGAATTG
ACTCGATCC
crtE-3_2-rev
CACAGGAGTCCTCACCCCAGGTCCAGAGAGAAACGGTGCAGGCAATCACGCGCTTCAGAGGACGC
GTTCGAATTGACTCGATCC
crtE-3_3-rev
CACAGGAGTCCTCACCGGTGTCGGTCATGCCATCGGTCAGATCGTCCAGCAGCTGGAACGCCTGGG
AATTGACTCGATCC
crtE-3_4-rev
CACAGGAGTCCTCACCACGCGGACCCAGCAGGTTAACCAGGGTAGATTTACCCGCGTCCTGGTTAG
AATCTTTGCGAATTGACTCGATCC
crtE-3_5-rev
CACAGGAGTCCTCACCAGACAGGTGTTCAGACGCCAGCTGCAGGTGCTGACGCAGACGTTCTTCAA
CCGGAATTGACTCGATCC
crtE-3_6-rev
CACAGGAGTCCTCACGCCTGGATGAAGTGCTGGGTCGCGTGGCCGTGCTGGCACGCCGGAATTGAC
TCGATCC
crtE-3_7-rev
CACAGGAGTCCTCACCGAGATAGAAGACAGTTAAGAAACCGCCGCCAGTTTTTTATCGAACCACGA
ATTGACTCGATCC

crtB-1
crtB-1_1-for
GTATAGAGTCAGTCGACGCTCTGAAGACCCATGGAAGTCGGTTCCAAGTCCTTCGCGACGGCGTCT
AAGCTGTTCGACGCGAAAACATACAGACTCGACAG

crtB-1_2-for
GTATAGAGTCAGTCGGCGCCGCTCCGTTCTGATGCTGTACGCGTGGTGCCGCCACTGCGATACAGA
CTCGACAG
crtB-1_3-for
GTATAGAGTCAGTCGACGACGTGATCGACGACCAGGTGCTGGGCTTCTCCAATGACACCCCGTCCAT
ACAGACTCGACAG
crtB-1_4-for
GTATAGAGTCAGTCGCTGCAATCCGCTGAGCAGCGCCTGGCCCAACTGGAAATGAAAACGCGCCAT
ACAGACTCGACAG
crtB-1_5-for
GTATAGAGTCAGTCGAGGCTTACGCGGGCTCTCAGATGCACGAACCGGCGTTCGCGGCCATACAGA
CTCGACAG
crtB-1_6-for
GTATAGAGTCAGTCGTTCCAAGAAGTGGCGATGGCTCACGACATCCTGCCGGCCTACGCATTTGACC
ATCCTGTCTTCTATCTCGATACAGACTCGACAG
crtB-1_1-rev
GTATAGAGTCAGTCGTCGCGAAGGACTTGGAACCGACTTCCATGGGTCTTCAGAGCGTATACAGAC
TCGACAG
crtB-1_2-rev
GTATAGAGTCAGTCGGTACAGCATCAGAACGGAGCGGCGCGTTTTCGCGTCGAACAGCTTAGACGC
CGATACAGACTCGACAG
crtB-1_3-rev
GTATAGAGTCAGTCGAGCACCTGGTCGTCGATCACGTCGTCGCAGTGGCGGCACCACGCATACAGA
CTCGACAG
crtB-1_4-rev
GTATAGAGTCAGTCGAGGCGCTGCTCAGCGGATTGCAGGGACGGGGTGTCATTGGAGAAGCCCAT
ACAGACTCGACAG
crtB-1_5-rev
GTATAGAGTCAGTCGTGCATCTGAGAGCCCGCGTAAGCCTGGCGCGTTTTCATTTCCAGTTGGGCCA
TACAGACTCGACAG
crtB-1_6-rev
GTATAGAGTCAGTCGATGTCGTGAGCCATCGCCACTTCTTGGAAGGCCGCGAACGCCGGTTCGATA
CAGACTCGACAG
crtB-1_7-rev
GTATAGAGTCAGTCGCGAGATAGAAGACAGGATGGTCAAATGCGTAGGCCGGCAGGATACAGACT
CGACAG

crtB-2
crtB-2_1-for
GTATAGAGTCAGTCGACGCTCTGAAGACCCCCGGCCTACGCATTTGACCATCTGGCCGGTTTCGCGA
TGGACGTGCATGAAACCCGAGACGACTCGTAAG

crtB-2_2-for
GTATAGAGTCAGTCGGCTACCAGACGCTGGACGACACCCTGCGCTACTGCTACCACGTCGCTGGTGT
GGAGACGACTCGTAAG
crtB-2_3-for
GTATAGAGTCAGTCGGTCGGCCTGATGATGGCGCAGATCATGGGTGTGCGTGACAACGCCACTCTG
GATCGGAGACGACTCGTAAG
crtB-2_4-for
GTATAGAGTCAGTCGTGCGTGCGACCTGGGTCTGGCGTTCCAGCTGACCAACATCGCCCGCGGAGA
CGACTCGTAAG
crtB-2_5-for
GTATAGAGTCAGTCGACATCGTAGAGGACGCCGAAGCAGGTCGTTGCTACCTGCCTGCGGCATGGC
TGGAGACGACTCGTAAG
crtB-2_6-for
GTATAGAGTCAGTCGGCCGAAGAAGGTCTGACGCGCGAGAATCTGGCGGACCCACAGAATCGCAA
GGCTCCTGTCTTCTATCTCGGAGACGACTCGTAAG
crtB-2_1-rev
GTATAGAGTCAGTCGGGCCAGATGGTCAAATGCGTAGGCCGGGGGTCTTCAGAGCGTGAGACGAC
TCGTAAG
crtB-2_2-rev
GTATAGAGTCAGTCGCAGGGTGTCGTCCAGCGTCTGGTAGCGGGTTTCATGCACGTCCATCGCGAA
ACCGAGACGACTCGTAAG
crtB-2_3-rev
GTATAGAGTCAGTCGCCATGATCTGCGCCATCATCAGGCCGACCACACCAGCGACGTGGTAGCAGT
AGCGGAGACGACTCGTAAG
crtB-2_4-rev
GTATAGAGTCAGTCGCGCCAGACCCAGGTCGCACGCACGATCCAGAGTGGCGTTGTCACGCACACG
AGACGACTCGTAAG
crtB-2_5-rev
GTATAGAGTCAGTCGCGACCTGCTTCGGCGTCCTCTACGATGTCGCGGGCGATGTTGGTCAGCTGG
AAGAGACGACTCGTAAG
crtB-2_6-rev
GTATAGAGTCAGTCGAGATTCTCGCGCGTCAGACCTTCTTCGGCCAGCCATGCCGCAGGCAGGTAG
CAAGAGACGACTCGTAAG
crtB-2_7-rev
GTATAGAGTCAGTCGCGAGATAGAAGACAGGAGCCTTGCGATTCTGTGGGTCCGCCGAGACGACTC
GTAAG

crtB-3
crtB-3_1-for
GTATAGAGTCAGTCGACGCTCTGAAGACCCGACCCACAGAATCGCAAGGCTCTGTCCCGTGTTGCTC
GCCGTCTGGTGGATCGGACTCGTACC

crtB-3_2-for
GTATAGAGTCAGTCGGAAACCGCGGAGCCATACTACCGCAGCGCGTCCGCAGGTCTGCGATCGGAC
TCGTACC
crtB-3_3-for
GTATAGAGTCAGTCGCTGGTCTGCCTCTGCGCTCTGCTTGGGCAATCGCAACGGCGCAGATCGGACT
CGTACC
crtB-3_4-for
GTATAGAGTCAGTCGGCAAGTGTACCGCAAGATCGGCATGAAGGTCGTGCAGGCGGGTTCCCGATC
GGACTCGTACC
crtB-3_5-for
GTATAGAGTCAGTCGAGGCGTGGGAACAGCGCCAAAGCACCTCCACGCCGGAAAAGCTGGGATCG
GACTCGTACC
crtB-3_6-for
GTATAGAGTCAGTCGCACTGCTGGTTGCTGCCAGCGGTCAAGCCGTGACCTCTCGCGTAGGATCGG
ACTCGTACC
crtB-3_7-for
GTATAGAGTCAGTCGCCCGTCATGCGCCTCGTTCTGCGGATCTGTGGCAGCGTCCGGTTTAACTGTC
TTCTATCTCGGATCGGACTCGTACC
crtB-3_1-rev
GTATAGAGTCAGTCGGACAGAGCCTTGCGATTCTGTGGGTCGGGTCTTCAGAGCGTGATCGGACTC
GTACC
crtB-3_2-rev
GTATAGAGTCAGTCGGCGGTAGTATGGCTCCGCGGTTTCCACCAGACGGCGAGCAACACGGGATCG
GACTCGTACC
crtB-3_3-rev
GTATAGAGTCAGTCGGCAGAGCGCAGAGGCAGACCAGGCAGACCTGCGGACGCGCTGATCGGACT
CGTACC
crtB-3_4-rev
GTATAGAGTCAGTCGTTCATGCCGATCTTGCGGTACACTTGCTGCGCCGTTGCGATTGCCCAAGATC
GGACTCGTACC
crtB-3_5-rev
GTATAGAGTCAGTCGCTTTGGCGCTGTTCCCACGCCTGGGAACCCGCCTGCACGACCGATCGGACTC
GTACC
crtB-3_6-rev
GTATAGAGTCAGTCGCGCTGGCAGCAACCAGCAGTGCCAGCTTTTCCGGCGTGGAGGTGGATCGGA
CTCGTACC
crtB-3_7-rev
GTATAGAGTCAGTCGGCAGAACGAGGCGCATGACGGGCTACGCGAGAGGTCACGGCTTGACGATC
GGACTCGTACC
crtB-3_8-rev
GTATAGAGTCAGTCGCGAGATAGAAGACAGTTAAACCGGACGCTGCCACAGATCCGATCGGACTCG
TACC

crtI-1

crtI-1_1-for

CCAAGGAGTCGCTAGACGCTCTGAAGACCCATGAACCGTACGACCGTAATTGGTGCGGGCTTCGGT
GGCCTGGCACTGGCGGAAGGGACTCGTACG

crtI-1_2-for

CCAAGGAGTCGCTAGATCCGTCTGCAAGCCTCTGGTGTCCCTACTCGTCTGCTGGAACAGCGCGACA
GAAGGGACTCGTACG

crtI-1_3-for

CCAAGGAGTCGCTAGAACCAGGCGGTCGTGCTTACGTGTACCAAGATCAGGGTTTCACCTTCGACG
CTGGTGAAGGGACTCGTACG

crtI-1_4-for

CCAAGGAGTCGCTAGCCGACTGTCATCACCGATCCATCTGCGATCGAAGAACTGTTTACTCTGGCCG
GCAAGAAGAAGGGACTCGTACG

crtI-1_5-for

CCAAGGAGTCGCTAGGCTGTCTGACTACGTGGAACTGATGCCAGTTAAACCATTTTATCGTCTGTGC
TGGGAATCTGGTAAGGTATTGAAGGGACTCGTACG

crtI-1_6-for

CCAAGGAGTCGCTAGCAGCTACGACAACGATCAACCGGCCCTGGAAGCGCAGATTGCGGCCTTCAA
CCGAAGGGACTCGTACG

crtI-1_7-for

CCAAGGAGTCGCTAGCGCGTGATGTAGAGGGCTATCGCCGTTCTGTCTTCTATCTCGGAAGGGACTC
GTACG

crtI-1_1-rev

CCAAGGAGTCGCTAGCCCGCACCAATTACGGTCGTACGGTTCATGGGTCTTCAGAGCGTGAAGGGA
CTCGTACG

crtI-1_2-rev

CCAAGGAGTCGCTAGGGGACACCAGAGGCTTGCAGACGGATCGCCAGTGCCAGGCCACCGAAGGA
AGGGACTCGTACG

crtI-1_3-rev

CCAAGGAGTCGCTAGGGTACACGTAAGCACGACCGCCTGGTTTGTCGCGCTGTTCCAGCAGACGAG
TAGAAGGGACTCGTACG

crtI-1_4-rev

CCAAGGAGTCGCTAGTCGCAGATGGATCGGTGATGACAGTCGGACCAGCGTCGAAGGTGAAACCC
TGATCTTGAAGGGACTCGTACG

crtI-1_5-rev

CCAAGGAGTCGCTAGACTGGCATCAGTTCCACGTAGTCAGACAGCTTCTTGCCGGCCAGAGTAAAC
AGTTCTTCGAGAAGGGACTCGTACG

crtI-1_6-rev

CCAAGGAGTCGCTAGAGGGCCGGTTGATCGTTGTCGTAGCTGAATACCTTACCAGATTCCCAGCACA
GACGATAAAATGGTTTAGAAGGGACTCGTACG

crtI-1_7-rev

CCAAGGAGTCGCTAGCGAGATAGAAGACAGAACGGCGATAGCCCTCTACATCACGCGGGTTGAAG
GCCGCAATCTGCGCTTCCGAAGGGACTCGTACG

crtI-2

crtI-2_1-for

CCAAGGAGTCGCTAGACGCTCTGAAGACCCGTGATGTAGAGGGCTATCGCCGTTTTCTGGCGTATTC
TCGCGCGGTCTTTGCGGAAGGTTAATACAGACTCGACAG

crtI-2_2-for

CCAAGGAGTCGCTAGCCTGAAGCTGGGCACTGTGCCATTCCTGTCTTTCCGTGACATGCTGCGTGCT
GCATACAGACTCGACAG

crtI-2_3-for

CCAAGGAGTCGCTAGACCGCAACTGGCTAAACTGCAGGCTTGGCGCTCCGTATATTCTAAAGTTGCG
TCTTACATCGAGATACAGACTCGACAG

crtI-2_4-for

CCAAGGAGTCGCTAGGACGAGCACCTGCGTCAAGCGTTCTCTTTTCACAGCCTGCTGGTCGGCGGAT
ACAGACTCGACAG

crtI-2_5-for

CCAAGGAGTCGCTAGTAACCCGTTTGCGACCTCTTCCATCTATACGCTGATCCACGCACTGGAACGC
GAGTGGATACAGACTCGACAG

crtI-2_6-for

CCAAGGAGTCGCTAGGGTGTGTGGTTCCCTCGTGGCGGTACGGGTGCCCTGGTGCAAGGCATATAC
AGACTCGACAG

crtI-2_7-for

CCAAGGAGTCGCTAGGGTAAAGCTGTTCGAGGATCTGGGTGGTGAAGTTGAGCTGAACGCGTCTGT
AGCTCGCCCTGTCTTCTATCTCGATACAGACTCGACAG

crtI-2_1-rev

CCAAGGAGTCGCTAGGCCAGAAAACGGCGATAGCCCTCTACATCACGGGTCTTCAGAGCGTATACA
GACTCGACAG

crtI-2_2-rev

CCAAGGAGTCGCTAGAGGAATGGCACAGTGCCCAGCTTCAGGTAACCTTCCGCAAAGACCGCGCGA
GAATACATACAGACTCGACAG

crtI-2_3-rev

CCAAGGAGTCGCTAGCAAGCCTGCAGTTTAGCCAGTTGCGGTGCAGCACGCAGCATGTCACGGAAA
GACATACAGACTCGACAG

crtI-2_4-rev

CCAAGGAGTCGCTAGGAGAACGCTTGACGCAGGTGCTCGTCCTCGATGTAAGACGCAACTTTAGAA
TATACGGAGCGCATACAGACTCGACAG

crtI-2_5-rev

CCAAGGAGTCGCTAGAGCGTATAGATGGAAGAGGTCGCAAACGGGTTACCGCCGACCAGCAGGCT
GTGAAAAATACAGACTCGACAG

crtI-2_6-rev

CCAAGGAGTCGCTAGCCGCCACGAGGGAACCACACACCCCACTCGCGTTCCAGTGCGTGGATCATA
CAGACTCGACAG

crtI-2_7-rev

CCAAGGAGTCGCTAGCTTCACCACCCAGATCCTCGAACAGCTTTACCATGCCTTGCACCAGGGCACC CGTAATACAGACTCGACAG

crtI-2_8-rev

CCAAGGAGTCGCTAGCGAGATAGAAGACAGGGCGAGCTACAGACGCGTTCAGCTCAAATACAGAC TCGACAG

crtI-3

crtI-3_1-for

CCAAGGAGTCGCTAGACGCTCTGAAGACCCTGAACGCGTCTGTAGCTCGCCTGGAAACTCAAGAAA ACCGCATCACGGCAGTGCGATCGGACTCGTACC

crtI-3_2-for

CCAAGGAGTCGCTAGATCTGAAAGACGGTCGCGTGTTTCCAACTCGTGCAGTTGCCTCCAATGCCGA CGGATCGGACTCGTACC

crtI-3_3-for

CCAAGGAGTCGCTAGTGGTTCATACTTATCGTGAGCTGCTGTCTCAACACCCTGCTTCTCAGGCCCA GGGTCGATCGGACTCGTACC

crtI-3_4-for

CCAAGGAGTCGCTAGGCTCTCTGCAAAACAAGCGTATGTCTAATAGCCTGTTTGTAATTTACTTCGG CCTGAACCATCACCACGATCGATCGGACTCGTACC

crtI-3_5-for

CCAAGGAGTCGCTAGAGCTGGCGCACCACACCGTCTGTTTTGGCCCGCGCTACCGCGAGATCGGAC TCGTACC

crtI-3_6-for

CCAAGGAGTCGCTAGGCTGATTGACGAAATCTTCAACAAAGATGGTCTGGCGGAGGATTTCTCTCT GTACCTGCACGCTCGATCGGACTCGTACC

crtI-3_7-for

CCAAGGAGTCGCTAGCTTGCGTTACTGACCCGTCCCTGGCCCCTGAGGGCTGCGGCAGCTCTGTCTT CTATCTCGGATCGGACTCGTACC

crtI-3_1-rev

CCAAGGAGTCGCTAGTTCCAGGCGAGCTACAGACGCGTTCAGGGTCTTCAGAGCGTGATCGGACTC GTACC

crtI-3_2-rev

CCAAGGAGTCGCTAGGAGTTGGAAACACGCGACCGTCTTTCAGATGCACTGCCGTGATGCGGTTTT CTTGAGTGATCGGACTCGTACC

crtI-3_3-rev

CCAAGGAGTCGCTAGTGTTGAGACAGCAGCTCACGATAAGTATGAACCACGTCGGCATTGGAGGCA ACTGCACGATCGGACTCGTACC

crtI-3_4-rev

CCAAGGAGTCGCTAGAACAGGCTATTAGACATACGCTTGTTTTGCAGAGAGCGACCCTGGGCCTGA GAAGCAGGGGATCGGACTCGTACC

crtI-3_5-rev

CCAAGGAGTCGCTAGCAGACGGTGTGGTGCGCCAGCTGATCGTGGTGATGGTTCAGGCCGAAGTA AATTACAGATCGGACTCGTACC

crtI-3_6-rev

CCAAGGAGTCGCTAGGCCAGACCATCTTTGTTGAAGATTTCGTCAATCAGCTCGCGGTAGCGCGG CCAAAAGATCGGACTCGTACC

crtI-3_7-rev

CCAAGGAGTCGCTAGGCCAGGGACGGGTCAGTAACGCAAGGAGCGTGCAGGTACAGAGAGAAAT CCTCCGATCGGACTCGTACC

crtI-3_8-rev

CCAAGGAGTCGCTAGCGAGATAGAAGACAGAGCTGCCGCAGCCCTCAGGGGATCGGACTCGTACC

crtI-4

crtI-4_1-for

CCAAGGAGTCGCTAGACGCTCTGAAGACCCCTGAGGGCTGCGGCAGCTACTACGTCCTGGCTCCTG TTCCGCACCTGGGGTAGAGACTCGATCC

crtI-4_2-for

CCAAGGAGTCGCTAGTACGGCGGACATCGACTGGGCGGTTGAGGGTCCGCGCCTGCGTGAGTAGA GACTCGATCC

crtI-4_3-for

CCAAGGAGTCGCTAGCCGCATTTTCGACTATCTGGAGCAGCACTACATGCCTGGCCTGCGCAGCCAA CTGGGTAGAGACTCGATCC

crtI-4_4-for

CCAAGGAGTCGCTAGTGACCCACCGTATTTTCACGCCATTCGATTTTCGTGACGAACTGAATGCCTAC CAGGGTTCTGCGTAGAGACTCGATCC

crtI-4_5-for

CCAAGGAGTCGCTAGCTTTTCCGTTGAACCAATTCTGACCCAAAGCGCATGGTTCCGTCCGCACAAT CGTGACAAGAACATTAAGTAGAGACTCGATCC

crtI-4_6-for

CCAAGGAGTCGCTAGCAACCTGTATCTGGTTGGTGCCGGTACGCACCCAGGCGCCGGTATTCCGGG GTAGAGACTCGATCC

crtI-4_7-for

CCAAGGAGTCGCTAGCGTTATTGGCTCTGCGAAAGCGACCGCGGGTCTGATGCTGGAGGACCTGGC CTAACTGTCTTCTATCTCGGTAGAGACTCGATCC

crtI-4_1-rev

CCAAGGAGTCGCTAGACGTAGTAGCTGCCGCAGCCCTCAGGGGTCTTCAGAGCGTGTAGAGACTCG ATCC

crtI-4_2-rev

CCAAGGAGTCGCTAGCCGCCCAGTCGATGTCCGCCGTACCCAGGTGCGGAACAGGAGCCAGGGTA GAGACTCGATCC

crtI-4_3-rev

CCAAGGAGTCGCTAGCATGTAGTGCTGCTCCAGATAGTCGAAAATGCGGTCACGCAGGCGCGGACC CTCAAGTAGAGACTCGATCC

crtI-4_4-rev

CCAAGGAGTCGCTAGCGAAAATCGAATGGCGTGAAAATACGGTGGGTCACCAGTTGGCTGCGCAG GCCAGGGTAGAGACTCGATCC

crtI-4_5-rev

CCAAGGAGTCGCTAGGCGCTTTGGGTCAGAATTGGTTCAACGGAAAAGGCAGAACCCTGGTAGGC ATTCAGTTCGTCAGTAGAGACTCGATCC

crtI-4_6-rev

CCAAGGAGTCGCTAGGCGTACCGGCACCAACCAGATACAGGTTGTTAATGTTCTTGTCACGATTGTG CGGACGGAACCATGTAGAGACTCGATCC

crtI-4_7-rev

CCAAGGAGTCGCTAGGCGGTCGCTTTCGCAGAGCCAATAACGCCCGGAATACCGGCGCCTGGGTGT AGAGACTCGATCC

crtI-4_8-rev

CCAAGGAGTCGCTAGCGAGATAGAAGACAGTTAGGCCAGGTCCTCCAGCATCAGACCCGTAGAGAC TCGATCC

Supplementary references

1. Martin, V.J., Pitera, D.J., Withers, S.T., Newman, J.D. and Keasling, J.D. (2003) Engineering a mevalonate pathway in Escherichia coli for production of terpenoids. *Nature biotechnology*, **21**, 796-802.
2. Datsenko, K.A. and Wanner, B.L. (2000) One-step inactivation of chromosomal genes in Escherichia coli K-12 using PCR products. *P Natl Acad Sci USA*, **97**, 6640-6645.
